# Combined computational modeling and experimental analysis integrating chemical and mechanical signals suggests possible mechanism of shoot meristem maintenance

**DOI:** 10.1371/journal.pcbi.1010199

**Published:** 2022-06-21

**Authors:** Mikahl Banwarth-Kuhn, Kevin Rodriguez, Christian Michael, Calvin-Khang Ta, Alexander Plong, Eric Bourgain-Chang, Ali Nematbakhsh, Weitao Chen, Amit Roy-Chowdhury, G. Venugopala Reddy, Mark Alber

**Affiliations:** 1 Interdisciplinary Center for Quantitative Modeling in Biology, University of California, Riverside, California, United States of America; 2 Department of Applied Mathematics, University of California, Merced, California, United States of America; 3 Department of Mathematics, University of California, Riverside, California, United States of America; 4 Department of Botany and Plant Sciences, University of California, Riverside, California, United States of America; 5 Center for Plant Cell Biology, University of California, Riverside, California, United States of America; 6 Institute for Integrative Genome Biology, University of California, Riverside, California, United States of America; 7 Computer Science and Engineering Department, University of California, Riverside, California, United States of America; 8 Department of Electrical and Computer Engineering, University of California, Riverside, California, United States of America; University of California Irvine, UNITED STATES

## Abstract

Stem cell maintenance in multilayered shoot apical meristems (SAMs) of plants requires strict regulation of cell growth and division. Exactly how the complex milieu of chemical and mechanical signals interact in the central region of the SAM to regulate cell division plane orientation is not well understood. In this paper, simulations using a newly developed multiscale computational model are combined with experimental studies to suggest and test three hypothesized mechanisms for the regulation of cell division plane orientation and the direction of anisotropic cell expansion in the corpus. Simulations predict that in the Apical corpus, WUSCHEL and cytokinin regulate the direction of anisotropic cell expansion, and cells divide according to tensile stress on the cell wall. In the Basal corpus, model simulations suggest dual roles for WUSCHEL and cytokinin in regulating both the direction of anisotropic cell expansion and cell division plane orientation. Simulation results are followed by a detailed analysis of changes in cell characteristics upon manipulation of WUSCHEL and cytokinin in experiments that support model predictions. Moreover, simulations predict that this layer-specific mechanism maintains both the experimentally observed shape and structure of the SAM as well as the distribution of WUSCHEL in the tissue. This provides an additional link between the roles of WUSCHEL, cytokinin, and mechanical stress in regulating SAM growth and proper stem cell maintenance in the SAM.

## Author summary

A population of stem cells located at the growing tip of plants are critical for continued growth of the stem and formation of all above-ground organs. These cells divide in specific planes resulting in the layered organization of the shoot apical meristem (SAM) stem cell niche. Through a combination of biologically-calibrated computational model simulations, transient genetic perturbation experiments, and quantitative image analysis, we test three possible mechanisms of the regulation of cell division plane orientation and the direction of anisotropic cell expansion in the SAMs of *Arabidopsis*. Our analysis suggests that the transcription factor-WUSCHEL, plant hormone cytokinin, and mechanical stress regulate patterns of cell expansion and cell division plane orientation in a layer-specific fashion to maintain the layered structure and shape of SAMs which are critical for stem cell homeostasis.

## Introduction

Deciphering how chemical signals and physical forces interact to regulate the overall size, shape, and organizational structure of a growing tissue is a central problem in the development of animals and plants. In contrast to their animal counterparts, plant cells are physically adhered to each other through their shared cell wall and do not move relative to one another during tissue growth and morphogenesis [1–7]. As such, the precise regulation of cell growth and division rates, polarization, and division plane orientation play critical roles in pattern formation and maintaining the size and shape of plant tissues. While recent studies suggest that cell shape and tensile forces alone are sufficient to explain patterns of cell division plane orientation in plant tissues [1, 8–10], most research in this area has been limited to the plant epidermis and does not consider the role of chemical signals in orienting the direction of anisotropic cell expansion and division plane orientation. In this paper, we explore the interplay of chemical signals and mechanical stress in regulating cell division plane orientation and the direction of anisotropic cell expansion in the corpus of the shoot apical meristem (SAM) of *Arabidopsis thaliana*, as it provides an ideal system for studying cell behavior in a morphogenetic and physiological context.

The mechanisms underlying cell division in plants have been studied extensively [1, 8, 9]. At the same time, past studies have primarily focused on shape and/or stress-based rules to predict cell division plane orientation in the epidermal L1 and L2 cell layers only. For example, Errera’s rule- which assumes that cells divide along the shortest new wall dividing the mother cell’s volume in half- has been shown to successfully predict cell division plane orientation in tissues with locally spherical shape and homogeneous growth, such as the tunica layers of the central zone of the distal portion of the SAM [1, 8, 11–13]. However, recent experiments and computational studies indicate that anisotropic stress arising from heterogeneous growth (different between adjacent cells) and saddle-shaped regions of the SAM result in deviations to shape-based division rules [1, 9, 10, 14]. Louveaux et al. [1] explained these deviations by proposing that new cell walls orient along the local maximum of tensile stress on the mother cell wall. In either case, patterns of cell surface expansion in the SAM give rise to changes in cell shape and tensile forces that could influence the positioning of new cell walls. The challenge is to understand how chemical regulators such as the transcription factor-WUSCHEL (WUS) and plant hormone cytokinin (CK) interact with mechanical stress to control cell division plane orientation and maintain the layered organization and shape of actively growing SAMs.

In this paper, results from our newly developed, biologically-calibrated, 2D, multiscale computational model are integrated with experimental studies and quantitative image analysis to hypothesize and test three separate mechanisms for how local mechanical cues and global chemical signals interact to maintain proper SAM shape and structure over time. Additionally, we demonstrate that a 2D model provides an appropriate approximation to test these hypothesized mechanisms. The paper is organized as follows. First, results from experimental studies where we manipulated the levels and spatial patterns of WUS and CK are presented to demonstrate that precise spatial regulation of these chemical signals is critical for maintaining proper size and shape of the SAM. Next, we present results demonstrating the effects of WUS and CK on cell division plane orientation in the central region of the SAM corpus. These two sets of experimental results are then used to suggest three hypothesized mechanisms for how WUS and CK interact with mechanically-transmitted cues to regulate cell growth and division behaviors. The first two mechanisms assume the sole function of WUS and CK in regulating the direction of anisotropic expansion of cells, while the placement of new cell walls during division is determined according to either Errera’s rule or local patterns of in-plane tensile stress on the cell wall. In contrast, the third mechanism assumes dual roles for WUS and CK in directly regulating both anisotropic cell expansion and cell division plane orientation.

**Fig 1:**
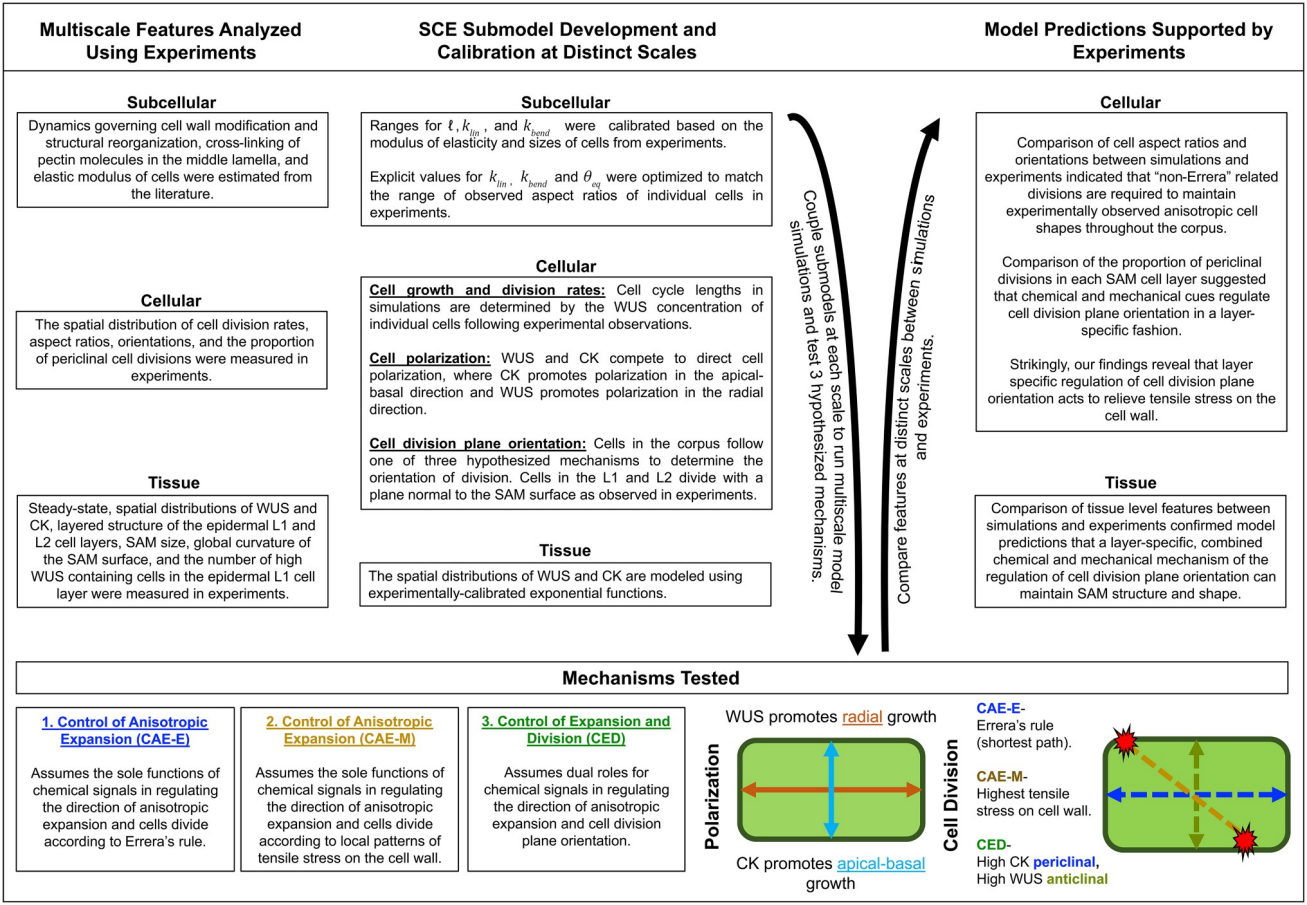
Combined multiscale modeling and experimental study workflow outline. Data from experimental studies was used to develop and calibrate submodel components at distinct scales. Multiscale model simulations were used to test three hypothesized mechanisms of the regulation of cell division plane orientation in the corpus. Results from perturbation experiments were used to support model predictions.

Next, we present results justifying the use of a two-dimensional (2D) model to simulate a longitudinal section of the central region of a growing SAM. Then, multiscale model simulations are used to test the three hypothesized mechanisms of SAM growth. Through direct comparison with experimental data, we arrive at our main modeling predictions; first, a combined chemical and mechanical mechanism regulates cell division plane orientation in a layer-specific fashion; second, this layer-specific, combined chemical and mechanical mechanism of regulation can maintain the experimentally observed shape and structure of the SAM as well as the distribution of WUS in the tissue. Next, we provide a detailed analysis of experimental studies upon manipulation of WUS and CK demonstrating that experimental results align with model simulation predictions. The paper ends with a Discussion section, where results are summarized and predictions of the model are put in a more general biological context. This section also describes future extensions of the computational modeling environment as well as its current limitations. A general description of the combined multiscale modeling and experimental analysis method we use is provided in Fig 1.

### Biological background

**Fig 2:**
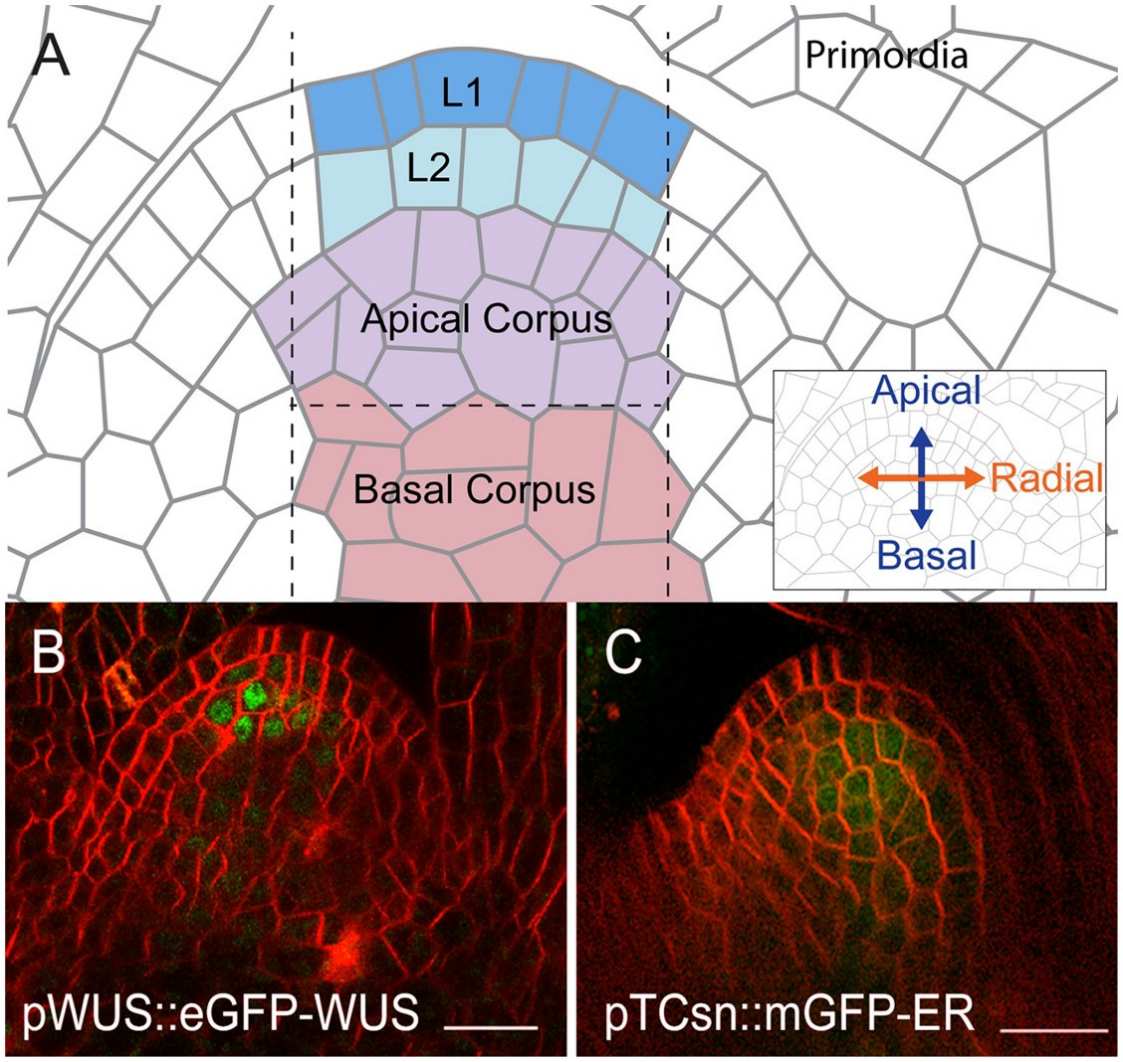
Organizational structure of the SAM. (A) Diagram showing a median longitudinal section of the SAM and depicting three distinct clonal layers. The tunica encompasses the L1 and L2 cell layers. The corpus is subdivided into the Apical corpus and Basal corpus. Vertical dashed lines represent the outer edges of the region used in experimental analysis. The horizontal dashed line represents the separation between the Apical corpus and Basal corpus. The L1 (blue), L2 (light blue), Apical corpus (purple), and Basal corpus (red) cells that fall within the region used for experimental analysis. These limits were manually determined for each experimental SAM image. (B) Median longitudinal section of the SAM showing the WUS protein domain *(pWUS::eGFP-WUS)* in a 9 day old SAM. (C) Median longitudinal section of the SAM showing the cytokinin signaling reporter *(pTCSn::mGFP5-ER)* in a 9 day old SAM. eGFP-WUS and mGFP5-ER (green) are overlaid on FM4–64 plasma membrane stain (Red). Scale bars = 20 *μ*m.

Located at the growing tip of the plant, the SAM maintains a set of stem cells that gradually divide and are pushed out laterally to differentiate and produce all above-ground organs, all while preserving its characteristic dome-like shape and multi-layered structure. Traditionally, SAMs have been divided into distinct clonal layers and zones [15–17] (Fig 2A and Fig A in S1 Appendix). The tunica consists of the outermost epidermal L1 layer and an inner sub-epidermal L2 layer. Both the L1 and L2 are composed of a single layer of cells that divide exclusively anticlinally (perpendicular to the SAM surface) ensuring that each layer remains one cell thick (Fig 2A). During development, the L3 cell layer, through periclinal cell divisions, forms into the multi-layered corpus, which we further separated into the Apical corpus and Basal corpus (Fig 2A). As such, rules governing the position of new cell walls are essential to maintain the clonally distinct layers, ensuring proper SAM organization during growth. While anticlines and periclines are traditionally used to quantify the patterning of division plane placement relative to the nearest tissue surface or sub-epidermal cell layers [18, 19], such definitions would present problems in the present work because we observe considerable variation in SAM shape between some of the more deformed mutant phenotypes (e.g. flat vs. enlarged meristems). Thus, for consistency across mutants, we define periclinal divisions in the corpus as those occurring perpendicular to the apical-basal axis of the SAM, and anticlinal divisions in the corpus as those occurring parallel to apical-basal axis (i.e. perpendicular to periclinal divisions) of the SAM [15] (see Fig 2A and S1 Appendix for details).

In addition to this layered organization, the SAM is also subdivided into four distinct functional zones (Fig A in S1 Appendix). The central zone (CZ) contains a set of stem cells that span the tunica and L3 cell layer. Within the CZ, stem cell progeny are pushed away laterally into the peripheral zone (PZ) where they begin expression of genes involved in differentiation. The organizing center (OC) and rib meristem (RM) consists of stem cell progeny located beneath the CZ. Cells in the RM span the Basal corpus and gradually differentiate along the apical-basal axis to form the stem of the plant. Amidst this process of constant displacement and subsequent differentiation, the relative numbers of cells in each zone are maintained, requiring precise spatial and temporal regulation of both SAM growth and gene expression [20].

Genetic analysis has revealed the importance of several chemical regulators in SAM growth and stem cell maintenance [21–28]. In particular, the homeodomain transcription factor (TF)- WUSCHEL (WUS) and the plant hormone cytokinin (CK) have been shown to regulate SAM size, shape, and the number of stem cells [24, 29–32]. *WUS* expression domain and size is confined to the OC in part through a negative feedback loop by CLAVATA3 which represses radial and apical expansion of *WUS* expression. In addition, a new regulatory loop has been proposed through CLE40, a PZ diffused signaling peptide, which maintains the WUS domain by promoting *WUS* expression through an unknown non-cell-autonomous signal [33]. The WUS protein migrates into adjacent cells to form a concentration gradient (Fig 2B and Fig A in S1 Appendix). The concentration gradient resulting from this regulation has been shown to stabilize, i.e. it maintains a steady-state distribution within the tissue, and it moves upward with the growing distal portion of the SAM [31, 34].

Additionally, CK is perceived by a family of histidine kinase receptors localized in the RM, thus restricting the CK response to these cells as revealed by the CK signaling reporter-pTCSn [35, 36] (Fig 2C and Fig A in S1 Appendix). CK signaling has been shown to stabilize the WUS protein thus regulating the WUS gradient [31]. Consistent with this observation, ectopic activation of CK signaling results in taller SAMs [31]. Despite the central importance of WUS and CK in regulating SAM growth and stem cell maintenance, their precise roles and interaction in controlling cell division plane orientation in the RM is poorly understood.

## Results

### WUSCHEL and cytokinin signaling regulate SAM shape and size

**Fig 3:**
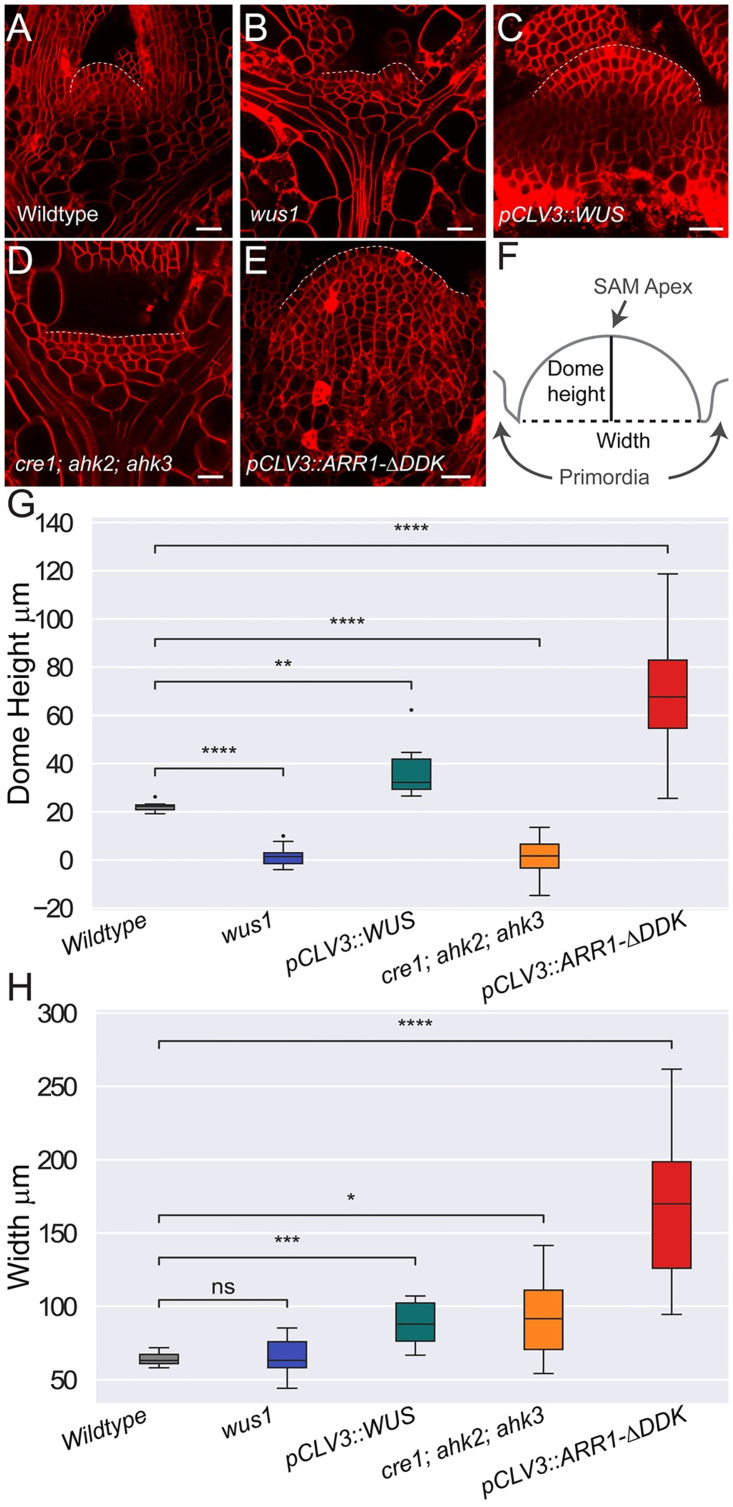
WUS and CK regulate SAM size and shape. Representative median longitudinal section of vegetative meristems in the (A) wildtype [l*er*], (B) *wus1–1*, and (D) *cre1–12;ahk2–2;ahk3–3* backgrounds. 48 hour Dex induction of (C) ectopic misexpression of WUS *[pCLV3::LhG4; 6xOP::eGFP-WUS-GR]* and (E) ectopic misexpression of CK *[pCLV3::LhG4; 6xOP::ARR1-*Δ*DDK-GR]* signaling. SAM dome has been outlined by white-dashed line. Scale bar = 20 microns. Image intensity and gain were increased in images (A,C, and D). (F) SAM dome height was measured from the primordia to the distal portion of the SAM and SAM width was measured from the apical junction of the nearest flanking primordia (see S3 Appendix for details). (G) Dome height and (H) width were determined for each experimental condition shown in (A-E). Significance tests in (G-H) were performed using independent t-tests. Asterisks indicate significance at the following levels: * (p ≤ 0.05), ** (p ≤ 0.01), *** (p ≤ 0.0001), **** (p ≤ 0.00001), ns (p > .05).

Both WUS and CK have been shown to influence shoot growth [29, 37]. To test the effects of WUS and CK on SAM size and shape, we analyzed the width and dome height of 7 to 10 day old SAMs upon manipulation of WUS and CK in experiments (Fig 3). First, we found that the dome height of *wus-1* null mutants and CK receptor mutants was significantly smaller than wildtype SAMs (p-value = 4.38e-13 and 3.67e-6 respectively), indicating that WUS and CK are required for maintaining the dome-like shape of the SAM (Fig 3B, 3D, 3G and 3H). Next, we found that ectopic misexpression of WUS and CK in the CZ significantly increases both the height and width of SAMs compared to wildtype (Fig 3C, 3E, 3G and 3H). Taken together, these results show that the precise spatial regulation of WUS and CK is critical for maintaining proper size and shape of the SAM.

### WUSCHEL inhibits periclinal cell divisions

**Fig 4:**
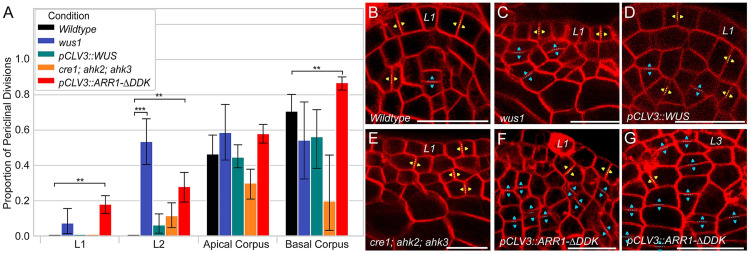
Layer-specific organization of cell division plane orientation. (A) Periclinal and anticlinal divisions were quantified by cell layer on a minimum of 10 SAMs (see S1 Appendix for details). Significance tests were performed using independent t-tests. Asterisks indicate significance at the following levels: ** (p ≤ 0.01), *** (p ≤ 0.0001). Higher magnification median longitudinal section from Fig 2 showing (B) wildtype [l*er*], (C) *wus1–1*, and (E) *cre1–12;ahk2–2;ahk3–3* vegetative meristems. 48 hour Dex induction of (D) ectopic misexpression of WUS *[pCLV3::LhG4; 6xOP::eGFP-WUS-GR]* and (F) ectopic misexpression of CK signaling *[pCLV3::LhG4; 6xOP::ARR1*-Δ*DDK-GR]*. (G) Characteristic “strips” of cells formed by repeated periclinal divisions in the Basal corpus of ectopic misexpression of CK experiments. (B-G) Positioning of new cell walls between speculated daughter cells after recent divisions are annotated in yellow arrowheads for anticlinal cell division and cyan arrowheads for periclinal cell division. Scale bar = 20 *μ*m.

In agreement with previous findings, we observed that cells in the epidermal L1 and L2 cell layers of wildtype SAMs divide exclusively in the anticlinal orientation (Fig 4A and 4B) [38, 39]. However, the *wus-1* mutants revealed the occurrence of periclinal cell divisions in both the L1 and L2 cell layers (Fig 4A and 4C). Our quantification also revealed a slight increase in the number of periclinal cell divisions in the Apical corpus of *wus-1* mutants compared to wildtype (Fig 4A). Although we detected changes in cell division patterns in the Basal corpus, the *wus-1* mutants produce very few cells in this region, resulting in high levels of noise and lack of sufficient data to statistically compare loss of function mutants alone. Thus, we further analyzed the impact of WUS on cell division plane orientation using ectopic misexpression experiments. It has been shown that ectopic misexpression of WUS in the CZ results in extremely low-level accumulation of the protein leading to an enlarged SAM [40]. In these SAMs, our analysis revealed the appearance of periclinal cell divisions in the L2 cell layer. This suggests a correlation between high WUS concentration and the inhibition of periclinal cell divisions (Fig 4A and 4D).

### Cytokinin promotes periclinal cell divisions

We found that the CK receptor mutants also revealed the occurrence of periclinal cell divisions in the L2 cell layer as well as a slight decrease in the proportion of periclinal cell divisions in the Apical corpus (Fig 4A and 4E). The occurrence of periclinal cell divisions in the L2 cell layer could be due to a decrease in WUS protein levels because CK has been shown to stabilize the WUS protein and maintain the WUS gradient [31]. Surprisingly, the slight decrease in the proportion of periclinal cell divisions in the Apical corpus despite a severe decrease in WUS protein levels suggests an independent role for CK signaling in either promoting periclinal cell divisions or inhibiting anticlinal cell divisions. Similarly, CK receptor mutants produce very few cells in the Basal corpus. Thus, to distinguish between these two possible mechanisms, we ectopically overexpressed type B ARABIDOPSIS RESPONSE REGULATOR1 (ARR1), a transcription factor downstream of CK signaling pathway which activates CK signaling targets. In those enlarged SAMs, we found a statistically significant increase in the proportion of periclinal cell divisions in the L1, L2, and Basal corpus (p-value = 1.72e-3, 3.80e-3, and 6.65e-3 respectively), as well as the consequent appearance of “strips” of cells formed by repeated periclinal divisions in the Basal corpus (Fig 4F and 4G, and S1 Fig). This suggests a correlation between high CK concentration and the promotion of periclinal cell divisions.

### Experimental observations suggest three separate mechanisms for the regulation of cell division plane orientation and anisotropic cell expansion in the corpus

We hypothesize that our experimental results demonstrating the effects of WUS and CK on SAM size, shape, and cell division plane orientation could be due to their roles in regulating cell growth and division behaviors. However, exactly how WUS and CK interact with mechanically-transmitted cues to regulate cell division plane orientation is unclear. It may be the case that WUS and CK only regulate anisotropic expansion of cells, and the placement of new cell walls is a consequence of cell shape (i.e. Errera’s rule) or mechanical stress. Alternatively, it may be the case that WUS and CK directly regulate both anisotropic expansion of cells and cell division plane orientation. Nonetheless, since CK promotes WUS protein stability and affects the WUS gradient [31] and *WUS* transcription [30], it is experimentally difficult if not impossible to uncouple the effects of these two regulators in controlling cell division plane orientation and the direction of anisotropic expansion of cells.

#### Testing hypothesized mechanisms of the regulation of SAM growth using a computational model

**Fig 5:**
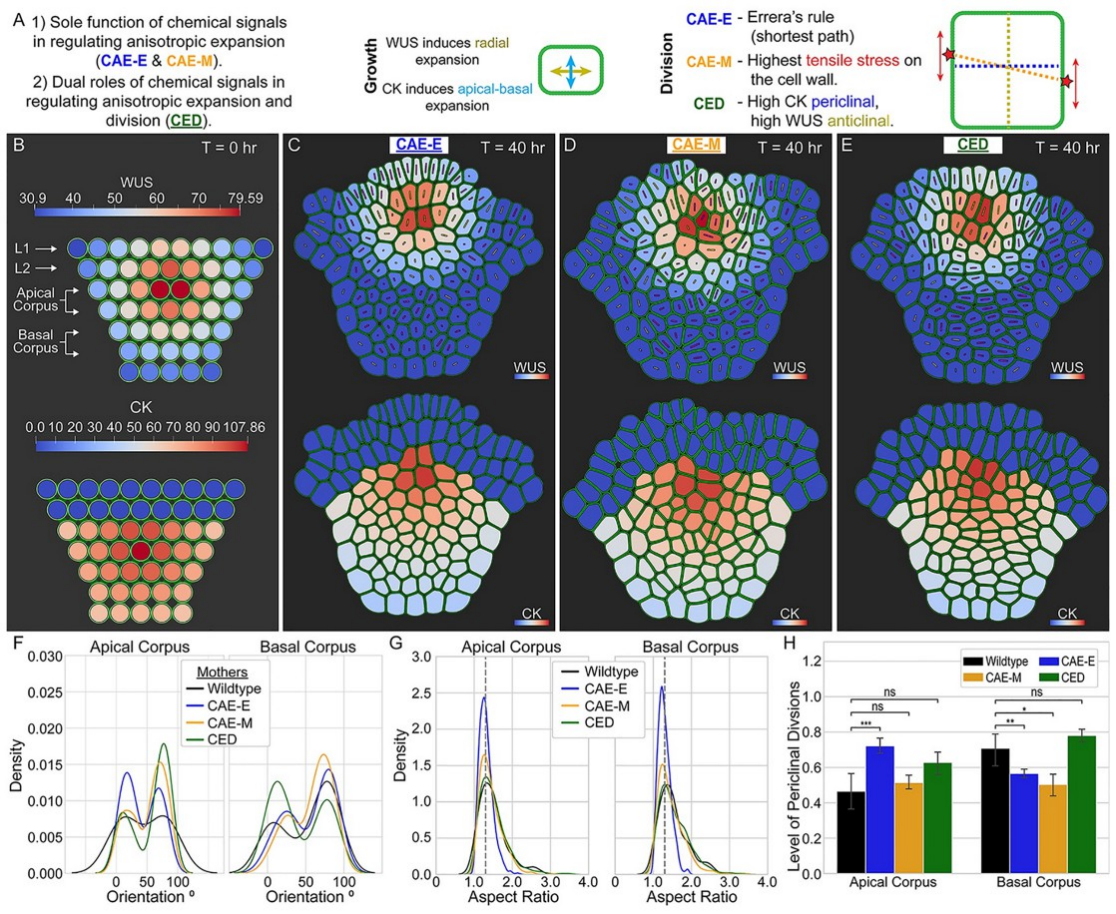
Results of computationally testing three hypothesized mechanisms of cell division plane orientation in the corpus. (A) Three hypothesized mechanisms for WUS and CK-mediated regulation of cell division plane orientation and the direction of anisotropic expansion of cells. (B) Cells are initialized as circles and allowed to “relax” into more biological cell shapes before growth and division begin (see S3 Appendix for details on model initial and boundary conditions). The internal colors of cells represent their levels of WUS (top row) and CK (bottom row). (C-E) Final simulation time point after 40 hours of growth reveals differences in cell shapes and orientations between each of the three mechanisms. The internal color shows the final levels and spatial patterns of WUS (top row) and CK (bottom row). Line segments inside cells are provided to help visualize cell aspect ratios and orientations. The length of each line segment is proportional to the encompassing cell’s aspect ratio, where cells with aspect ratio = 1 have line segments with length 0. The directional vector of each line segment represents the orientation of the longest axis of the encompassing cell. Purple lines denote daughter cells while yellow lines denote mother cells (see S1 Appendix for details on mother/daughter cell classification). (F) The distributions of mother cell orientations for the CAE-E (blue), CAE-M (gold), and CED (green) mechanisms were not statistically different from wildtype experiments (black) in both the Apical corpus (p-value = .5520, .6841, and .8330 respectively) and Basal corpus (p-value = .7567, .3103, and.2173 respectively). (G) The distributions of cell aspect ratios for the CAE-M (gold) and CED (green) mechanisms were not statistically different from wildtype experiments (black) in both the Apical corpus (p-value = .4879 and .9521 respectively) and Basal corpus (p-value = .1724 and .5781 respectively) while the CAE-E mechanism was significantly different (p-values < 1.0e-32 in both cell layers). (H) Proportion of periclinal cell divisions in the Apical and Basal corpus for all three mechanisms. In the Apical corpus, the CAE-M and CED mechanisms matched experiments, while the CAE-E mechanism did not (p-value = 2.93e-4). In the Basal corpus, only the CED mechanism matched experiments (p-value = 5.5e-3 and 3.86e-2 for the CAE-E and CAE-M mechanisms respectively). S2 Fig provides extended analysis between experimental wildtype SAMs and wildtype simulations for all four hypothesized mechanisms presented in this paper.

To address this, we developed a detailed, biologically calibrated, multiscale, computational model and used it to test three separate hypothesized mechanisms for how WUS, CK, and mechanical stresses can regulate cell division plane orientation in the corpus (for details see the “Model description” section and S3 Appendix). The first two mechanisms we tested assume that the sole functions of WUS and CK are in regulating the direction of anisotropic expansion of cells. In the model, the direction of anisotropic expansion of cells is determined by an experimentally-calibrated probability distribution where cells with relatively high levels of WUS are more likely to preferentially expand in the radial direction and cells with relatively high levels of CK are more likely to preferentially expand in the apical-basal direction (see the “Model description” section for details). We refer to this mechanism of regulation as “the control of anisotropic expansion” (CAE). In simulations assuming the CAE mechanism, we compared simulation output where division plane orientation was determined using either Errera’s rule (CAE-E mechanism), which assumes that cells divide by following the shortest path, or “the mechanical division rule,” (CAE-M mechanism), which assumes that cells divide according to maximum in-plane tensile stress on their cell wall (see Fig 5A and the “Model description” section for details).

The third mechanism we tested assumes dual roles for WUS and CK in both regulating anisotropic cell expansion and cell division plane orientation. We refer to this mechanism of regulation as “the control of expansion and division” (CED). In simulations assuming the CED mechanism, the direction of anisotropic cell expansion is determined by relative concentrations of WUS and CK as above, and cell division plane orientation is determined by a second experimentally-calibrated probability distribution where cells with relatively high levels of CK are more likely to divide periclinally (perpendicular to the root-to-shoot axis) and cells with relatively high levels of WUS are more likely to divide anticlinally (perpendicular to the SAM surface) (see Fig 5A and the “Model description” section for details).

To test the CAE-E, CAE-M and CED mechanisms described above, we simulated growing SAMs under three different experimental conditions where the levels and spatial patterns of WUS and CK were calibrated to be analogous with either wildtype, ectopic misexpression of WUS or ectopic misexpression of CK experiments (see the “Model description” section and S3 Appendix for details). We then compared model simulations directly with experiments using both cell (aspect ratios and orientations) and tissue level metrics (proportion of periclinal divisions, ratio of SAM width to SAM dome height, global curvature of the SAM surface, spatial distribution of WUS in the tissue and layered structure of the L1 and L2 cell layers) to determine the mechanism of WUS and CK-mediated regulation of cell division plane orientation in the corpus (see S1, S2 and S3 Appendices for biological relevance of the metrics we used and further details). Results below are based on 20 simulations for each of the nine conditions tested. Each simulation begins with an organized array of 50 cells in seven cell layers as shown in Fig 5B, and we allow each simulated meristem to grow for approximately 40 hours. Typical output from simulations involving each of the three mechanisms under wildtype conditions is shown in Fig 5B, 5C, 5D and 5E, and S1 Video. In what follows, we first demonstrate that a 2D model provides an appropriate approximation for studying cell behaviors in the central region of the corpus, and then we provide a detailed description of the model simulation results used to test the specific hypothesized mechanisms above.

#### Justification for using a 2D model combined with experimental data from 2D longitudinal SAM sections to study cell behaviors in the central region of the corpus

Our model simulates a two-dimensional (2D) longitudinal section of the central region of a growing SAM (as depicted in Fig 2A). To calibrate and validate our model, steady-state distributions of WUS and CK as well as specific cell level features (i.e. cell centroids, aspect ratios, and orientations) across individual cell layers were extracted from confocal microscopy images of longitudinal SAM sections (see S1 Appendix for details). In what follows, we present experimental results justifying our use of a 2D modeling framework to study cell behaviors in the central region of the corpus.

Experimentally observed symmetry in the WUS and CK signaling domains across SAM layers supports the application of a 2D model since it suggests that the apical half of the meristematic dome is radially symmetric, i.e. a longitudinal cut at any angle through the center of the meristem will give the same profile, with respect to the WUS and CK signaling domains within the central region of the SAM [22, 31, 34]. However, observing cell level features from single longitudinal sections could suffer from misinterpreting cell aspect ratios and orientations. This is especially true for smaller cells or cells whose longest axis occurs outside the longitudinal section being studied. Thus, in order to determine how well longitudinal section data captures the true distribution of cell aspect ratios and orientations in the SAM, we performed two additional analyses.

First, we compared the distributions of cell aspect ratios and orientations across 5–10 neighboring longitudinal sections for 9 wildtype SAMs (see S2 Appendix for details). When we restricted our analysis to cells lying within the central region of the corpus, we found no difference in the distributions of cell aspect ratios and orientations between adjacent longitudinal sections. Second, we used 3D reconstruction data to compare measurements from 3D cells to their corresponding 2D cell-sections. To be more specific, we measured the difference in degrees between an elongated cell’s longest axis and the apical-basal axis in 3D using reconstructed data, and performed the analogous measurement in 2D by taking the same cell’s longitudinal sections and measuring the angle their longest axis makes with the apical-basal axis (see S2 Appendix for details). Our analysis revealed that for 80% of cells, the difference between its measured orientation in 3D versus 2D was within 15°. Moreover, we observed that for elongated cells with aspect ratio greater than 1.3, there was a 91% match between the 3D and 2D categorization of a cell as being oriented in either the radial or apical-basal direction (see S2 Appendix for details). Additionally, by focusing our analysis on the central region of the corpus (depicted by vertical dashed lines in Fig 2A), we exclude the differentiation regions on the flanks that break radial symmetry to produce organ primordia. Taken together, these results indicate that the central region of the SAM can be approximated as a 2D “slice”, or longitudinal section. Moreover, using a 2D model allows us to simulate a large number of cells in very high detail, especially concerning the unique mechanical properties of plant cells (see the “Model description” section and S3 Appendix for more details).

#### Quantifying the direction of anisotropic cell expansion in the SAM

Expansion of plant cells is thought to result from cell walls yielding in response to turgor pressure [6]. Although turgor pressure is non-directional, heterogeneity in mechanical properties of the cell wall can lead to anisotropic cell expansion, i.e. cell expansion occurring preferentially in one axis over the other [41]. Individual cells in our model are assigned a preferred growth direction that is updated dynamically over the course of simulations according to their concentrations of WUS and CK (see the “Model description” section for details). However, cells in our simulations are also growing and dividing in a multicellular context; thus their resulting direction of anisotropic expansion is an emergent property that depends on local interactions with neighboring cells.

We used two main properties of cells to quantify the emergent direction of anisotropic cell expansion in simulations and experiments. First, we analyzed the distribution of cell aspect ratios to quantify the emergent degree of anisotropic cell expansion. Next, we analyzed the distribution of cell orientations to determine how WUS and CK impact the emergent direction of anisotropic cell expansion in the SAM. The orientation of an individual cell indicates the emergent direction of cell expansion relative to the tissue. For example, a cell with orientation equal to 0° is elongated in the radial direction, and a cell with orientation equal to 90° is elongated in the apical-basal direction. The aspect ratio and orientation of cells reported for all modeling results were computed directly from simulation data (see S3 Appendix for details). The aspect ratio and orientation of cells reported in experimental results were extracted from confocal microscopy images of median longitudinal sections of the SAM using the Image Processing Toolbox in MATLAB (see S1 Appendix for details). When comparing cell aspect ratios and orientations between simulations and experiments, we restricted our analysis to *elongated* cells which we defined to be cells that have aspect ratio > 1.3. This threshold was determined because the 2D anisotropic expansion direction of cells with aspect ratio > 1.3 more closely matches the cells they are taken from in 3D compared to cells with more isotropic (i.e. less elongated) shapes (see section “Justification for using a 2D model combined with experimental data from 2D longitudinal SAM sections to study cell behaviors in the central region of the corpus” and S2 Appendix for details).

#### WUSCHEL and cytokinin regulate the direction of anisotropic expansion of cells to maintain SAM structure in the corpus

To test whether it is possible that WUS and CK regulate the direction of anisotropic expansion of cells, we compared the distribution of mother cell orientations between wildtype experiments and simulations for the CAE-E, CAE-M, and CED mechanisms. We found that in the Apical and Basal corpus, all three mechanisms resulted in a distribution of mother cell orientations that was not significantly different from experiments (Fig 5F). Note that the distribution of mother cell orientations in the Apical corpus of wildtype experimental SAMs is more uniformly distributed between 0° and 90° compared to simulation results for all three mechanisms. This suggests that WUS and CK-mediated regulation of the direction of anisotropic expansion is sufficient to maintain experimentally observed patterns of cell growth throughout the corpus.

#### Non-Errera related divisions are important for production of anisotropic cell shapes in the corpus

We next sought out to investigate whether the CAE-E, CAE-M, and CED mechanisms could produce experimentally observed cell shapes in the corpus. To do this, we compared cell aspect ratios between simulations and experiments. In both the Apical and Basal corpus, we found that the CAE-M and CED mechanisms resulted in experimentally observed aspect ratios of cells, while the CAE-E mechanism did not (Fig 5G). Moreover, while the average aspect ratio of cells in CAE-M and CED simulations matched experiments, the average aspect ratio of cells in CAE-E simulations was significantly lower (p-value < 1.0e-32). This indicates that “non-Errera” related divisions are required to maintain experimentally observed anisotropic cell shapes throughout the corpus (Fig 5G).

#### CAE-M and CED mechanisms regulate cell division plane orientation in a layer-specific fashion

Next we tested whether the CAE-E, CAE-M, and CED mechanisms could produce experimentally observed proportions of periclinal divisions in the corpus. We found that in the Apical corpus, the CAE-M and CED mechanisms resulted in a proportion of periclinal divisions that was not significantly different from experiments, while the CAE-E mechanism was significantly higher (p-value = 2.93e-4) (Fig 5H). In contrast, in the Basal corpus, we found that only the CED mechanism resulted in a proportion of periclinal divisions that was not significantly different from experiments, while the CAE-E and CAE-M mechanisms were significantly lower (p-value = 5.5e-3 and 3.86e-2 respectively) (Fig 5H). Since both the CAE-M and CED mechanisms matched experimental cell shapes and the proportion of periclinal divisions in the Apical corpus, it is unclear which of these two mechanisms is likely to control cell division plane orientation there. However, since only the CED mechanism matched the proportion of periclinal divisions in the Basal corpus, this suggests that chemical and mechanical signals could regulate cell division plane orientation in a layer specific fashion. Thus, in order to uncouple the role of the CAE-M and CED mechanisms in directing cell division plane orientation in individual cell layers, we studied the effect of each mechanism in simulations where we independently perturbed the levels and spatial patterns of WUS and CK.

#### WUSCHEL and cytokinin directly regulate cell division plane orientation in the Basal corpus

**Fig 6:**
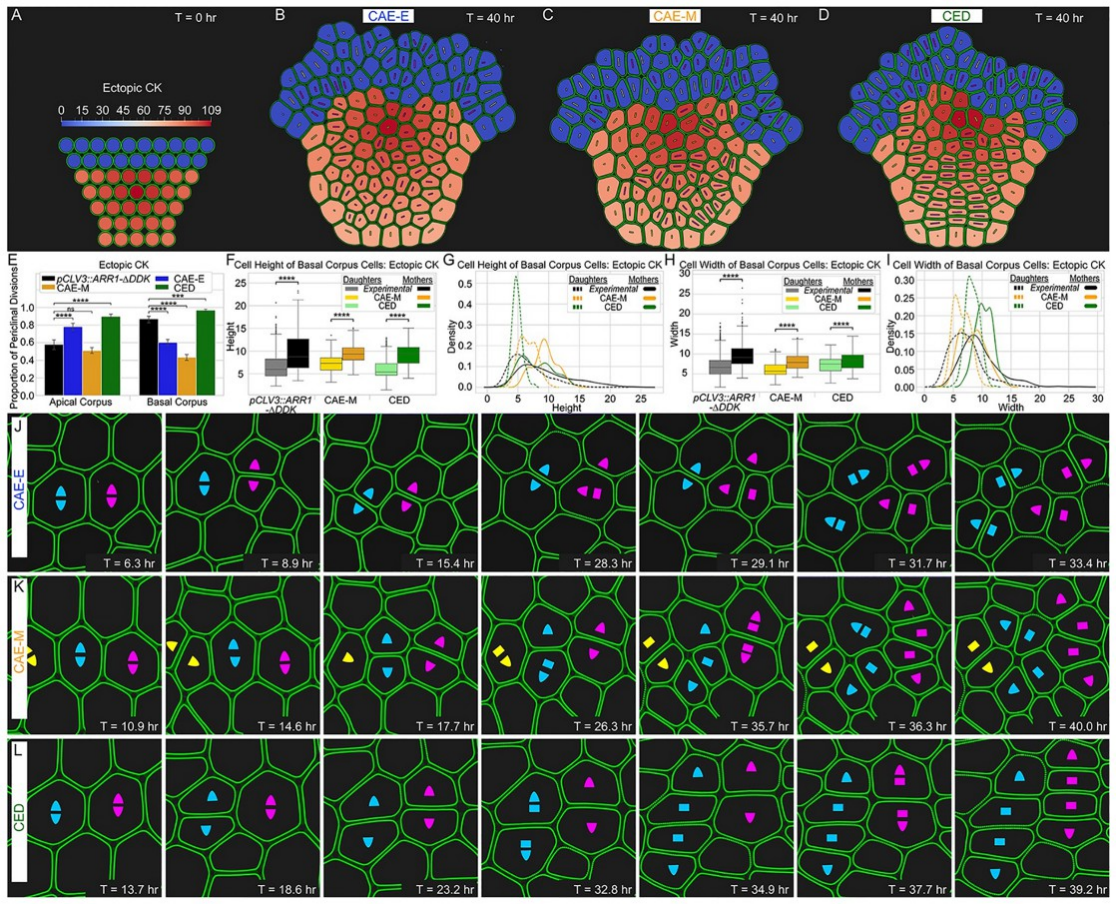
Quantitative comparison of experimental data with simulation results for ectopic misexpression of CK. (A) The spatial distribution of CK was expanded in the deeper layers to be analogous with ectopic misexpression of CK experiments (see the “Model description” section for details). (B-D) Final time point of ectopic CK simulations for all three mechanisms. (E) Proportion of periclinal divisions for three mechanisms compared to experiments in ectopic misexpression of CK simulations. Mother and daughter cell height (F) boxplots and (G) distributions for the CAE-M and CED mechanisms in ectopic CK simulations. Comparison of mother and daughter cell widths (H) boxplots and (I) distributions for the CAE-M and CED mechanisms in ectopic CK simulations. Significance tests in (F,H) were performed using independent t-tests. Asterisks indicate significance at the following levels: * (p ≤ 0.05), ** (p ≤ 0.01), *** (p ≤ 0.0001), **** (p ≤ 0.00001), ns (p > .05). (J) Time series of cell division and expansion events in CAE-E simulations. (K) Time series of cell division and expansion events in CAE-M simulations. (L) Time series of cell division and expansion events in CED simulations. CK induced increase in periclinal cell divisions coupled with CK induced apical-basal expansion of cells leads to the formation of characteristic “strips” in the Basal corpus. The CED mechanism is the only mechanism that can reproduce experimentally observed SAM growth patterns in this region. S3 Fig provides extended analysis between ectopic misexpression of CK SAMs in experiments and simulations for all four hypothesized mechanisms presented in this paper.

To better understand the mechanism controlling cell division plane orientation in the Basal corpus, we compared the effect of the CAE-M and CED mechanisms in simulations calibrated to be analogous with ectopic CK experiments. For these simulations, we expanded the spatial distribution of CK only, and we did not change the levels or spatial pattern of WUS (see Fig 6A, 6B, 6C and 6D, S2 Video, and the “Model description” section for details). We found that only the CED mechanism resulted in the three main properties observed in the Basal corpus of ectopic CK SAMs. First, the CED mechanism resulted in a drastic increase in the proportion of periclinal divisions between the Apical and Basal corpus- consistent with wildtype SAMs (Fig 6E). Second, the CED mechanism reproduced experimental observations regarding cell heights and widths. Namely, we found that the distribution of mother cell heights was statistically different from daughter cell heights (Fig 6F and 6G). We also found that the average width of mother cells was significantly bigger than daughter cells, and we observed no difference in their population variances-i.e. the distribution of mother cell widths appears as a “right-shifted copy” of the distribution of daughter cell widths consistent with experiments (Fig 6H and 6I). Third, the CED mechanism resulted in clearly defined “strips” commonly found in the Basal corpus of ectopic CK SAMs in experiments (Figs 4G and 6L, and S1 Fig). These results indicate that dual roles for WUS and CK in both regulating the direction of anisotropic cell expansion and cell division plane orientation is required to maintain the structure of the Basal corpus.

#### Apical corpus cells divide according to local patterns of maximum tensile stress on the cell wall

**Fig 7:**
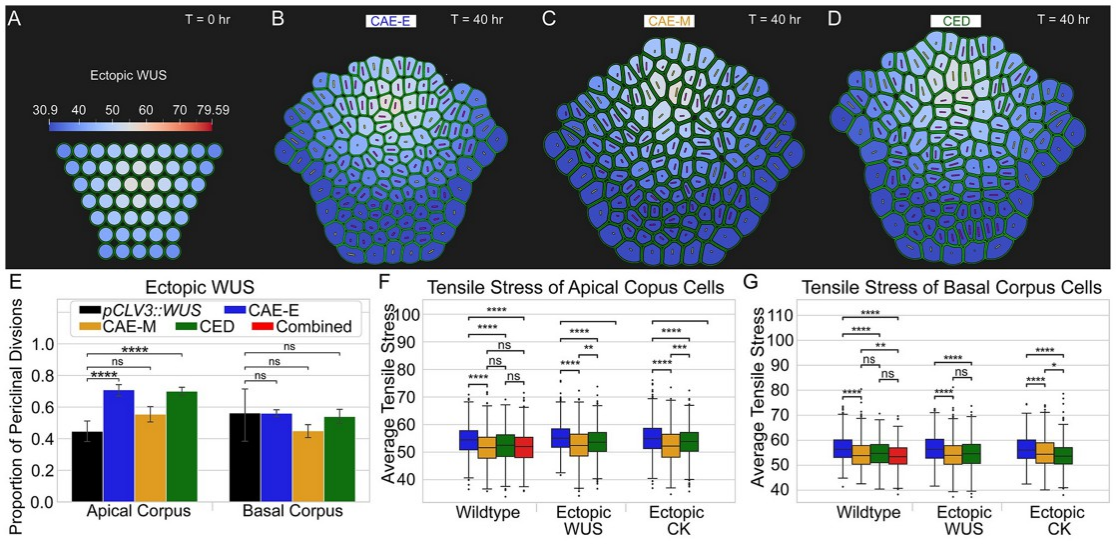
Quantitative comparisons of experimental data with simulation results for ectopic misexpression of WUS. (A) Initial levels and spatial pattern of WUS were calibrated to be analogous with ectopic misexpression of WUS experiments. The maximum WUS concentration was decreased to 2/3 wildtype concentrations and its spatial distribution was expanded in the corpus (see the “Model description” section for details). (B-D) Final time point of ectopic WUS simulations for all three mechanisms. (E) Proportion of periclinal divisions for three mechanisms compared to experiments in ectopic misexpression of WUS simulations. Average tensile stress on the cell wall for all three mechanisms in (F) the Apical corpus and (G) Basal corpus for wildtype, for wildtype, ectopic CK, and ectopic WUS simulations. Significance tests in (E-G) were performed using independent t-tests. Asterisks indicate significance at the following levels: * (p ≤ 0.05), ** (p ≤ 0.01), *** (p ≤ 0.0001), **** (p ≤ 0.00001), ns (p > .05). S4 Fig provides extended analysis between ectopic misexpression of WUS SAMs in experiments and simulations for all four hypothesized mechanisms presented in this paper.

To investigate the mechanism controlling cell division plane orientation in the Apical corpus, we compared the effect of the CAE-M and CED mechanisms in both ectopic CK simulations and simulations calibrated to be analogous with ectopic WUS experiments. We found that in ectopic CK simulations, only the CAE-M mechanism resulted in a proportion of periclinal divisions that was not statistically different from experiments. For ectopic WUS simulations, we decreased the maximum WUS level to 2/3 wildtype concentrations and expanded its spatial distribution consistent with experiments (see Fig 7A, 7B, 7C and 7D, S3 Video, and the “Model description” section for details). Similarly to ectopic CK simulations, we found that only the CAE-M mechanism resulted in a proportion of periclinal divisions that was not significantly different from experiments (Fig 7E). Thus, since the CAE-M mechanism was the only mechanism that resulted in the correct proportion of periclinal divisions in both ectopic CK and ectopic WUS simulations, these results further indicate the combined role of chemical and mechanical signals in predicting cell division plane orientation in the Apical corpus. In particular, our model simulations suggest the sole functions of WUS and CK are in regulating anisotropic cell expansion in the Apical corpus, and predict that cells divide according to local patterns of maximum in-plane tensile stress on their cell wall.

To further test this hypothesis, we quantified the average in-plane tensile stress on the cell wall for cells in CAE-E, CAE-M, and CED mechanisms in wildtype, ectopic WUS, and ectopic CK simulations. We found that in the Apical corpus, the CAE-M mechanism resulted in the minimum average tensile stress for both ectopic WUS and ectopic CK SAMs, while the CAE-M and CED mechanisms showed no difference for wildtype SAMs (Fig 7F). Furthermore, we found that in the Basal corpus, the CED mechanism resulted in the minimum average tensile stress on the cell wall in ectopic CK simulations, while the CAE-M and CED mechanisms were no different in wildtype and ectopic WUS simulations (Fig 7G). Strikingly, our findings reveal that layer specific regulation of cell division plane orientation acts to relieve in-plane tensile stress on the cell wall.

#### Layer-specific, combined chemical and mechanical regulation of cell division plane orientation can maintain SAM structure and shape

**Fig 8:**
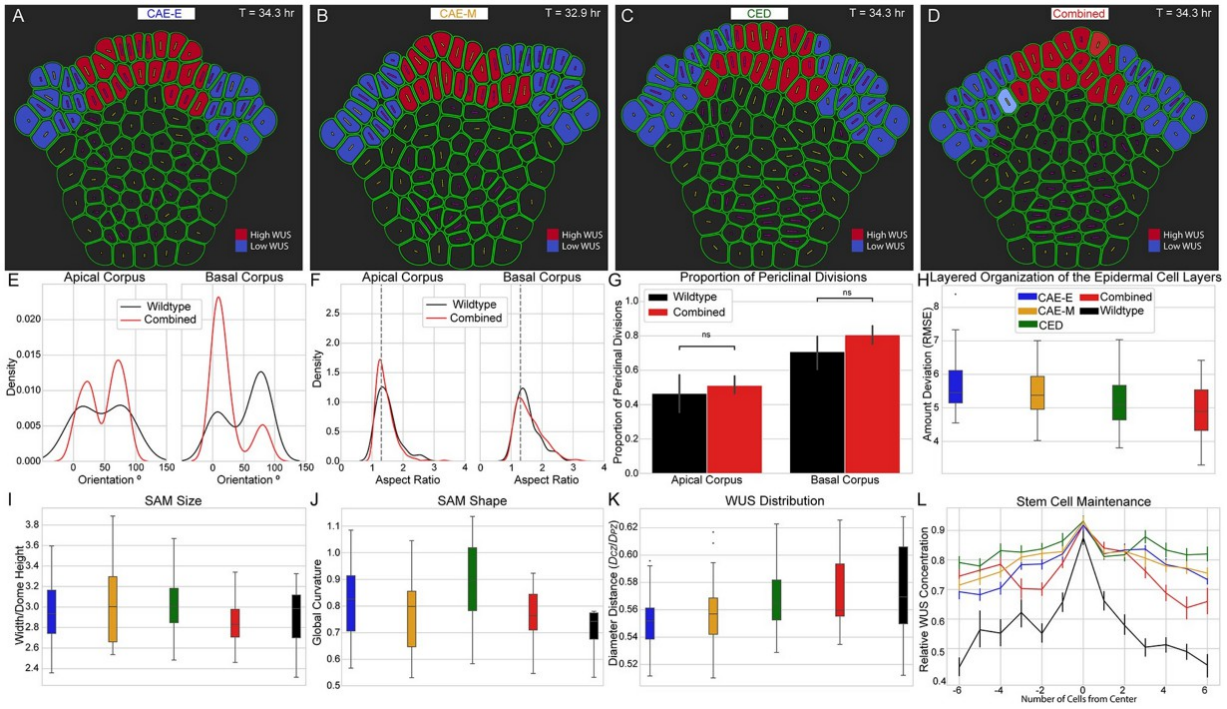
Layer-specific combined chemical and mechanical regulation of cell division plane orientation maintains proper shape, multi-layered structure and spatial distribution of WUS in the SAM. (A-D) Typical simulation output after 40 hours of growth for all four mechanisms (CAE-E, CAE-M, CED, and combined CAE-M and CED) in wildtype signaling conditions. Cell color highlights distinct patterns of WUS accumulation in the epidermal L1 and L2 cell layers for each mechanism (red = high WUS and blue = low WUS). (E-F) The combined CAE-M and CED mechanism resulted in distributions of (E) mother cell orientations that were similar to experiments and (F) cell aspect ratios that matched experiments. (G) The proportion of periclinal cell divisions in combined CAE-M and CED simulations were found to match experiments. (H) The combined CAE-M and CED mechanism resulted in the smallest amount of deviation from a single-cell layer in the epidermal L1 and L2 cell layers. The combined CAE-M and CED mechanism most closely matched experimentally observed SAM (I) size- the ratio of SAM width to dome height, (J) shape- global curvature of the SAM surface, and (K) WUS distribution in the SAM after 40 hrs of growth. (L) The combined CAE-M and CED mechanism resulted in the correct number of high WUS containing cells in the epidermal L1 cell layer. (E-L) Experimental wildtype (black), CAE-E (blue), CAE-M (gold), CED (green), and combined (red) in all panels. See S3 Appendix for detailed description of all metrics used in this Figure.

Based on our simulation results above (Figs 5–7), we next tested whether a layer-specific, combined chemical and mechanical mechanism regulating cell division plane orientation would maintain SAM structure and shape. To do this, we ran wildtype simulations where cells in the Apical corpus follow the CAE-M mechanism and cells in the Basal corpus follow the CED mechanism for division plane placement (Fig 8D). We refer to this model of regulation as “the combined CAE-M and CED” mechanism. First, we found that the combined CAE-M and CED mechanism resulted in a distribution of mother cell orientations that was not significantly different from experiments in the Apical corpus, and maintained a bimodal distribution in the Basal corpus (Fig 8E). Second, we found that the combined CAE-M and CED mechanism produced experimentally observed distribution of cell aspect ratios in both the Apical corpus and Basal corpus (Fig 8F). We also found that the proportion of periclinal divisions matched experiments in both the Apical corpus and Basal corpus (Fig 8G). Since we observed slightly more periclinal divisions in the Basal corpus of combined CAE-M and CED simulations compared to wildtype SAMs, we believe this could explain why the distribution of mother cell orientations in this region did not result in a direct match with the experimental distribution. Finally, we found that the combined CAE-M and CED mechanism resulted in the smallest amount of deviation from the layered organization of the epidermal L1 and L2 cell layers compared to the CAE-E, CAE-M, and CED mechanisms (Fig 8H). This suggests that a layer-specific, combined chemical and mechanical mechanism regulating cell division plane orientation is necessary to maintain the multi-layered structure of the SAM.

Next, we investigated whether the combined CAE-M and CED mechanism would result in the correct shape and size of the SAM. While all four mechanisms resulted in experimentally observed SAM size (i.e. average ratio of SAM width to dome height) (Fig 8I), our analysis revealed that the combined CAE-M and CED mechanism most closely matched experimentally observed SAM shape (i.e. global curvature) (Fig 8J). Notably, while both the CED and combined CAE-M and CED mechanisms most closely matched the experimentally observed distribution of WUS in the radial direction (Fig 8K), only the combined CAE-M and CED mechanism resulted in the correct number of high WUS containing cells in the epidermal L1 cell layer (Fig 8A–8D and 8L). S5 Fig provides additional analysis of the time evolution of simulated cell orientations and aspect ratios by condition and division plane mechanism.

### Detailed analysis of experimental studies upon manipulations of WUS and CK support model predictions

To test our model prediction that a layer-specific, combined chemical and mechanical mechanism regulating cell division plane orientation can maintain SAM structure and shape, we quantified the effect of WUS and CK on patterns of cell division plane orientation and anisotropic cell expansion in mutant and overexpression experiments. WUS accumulates at its highest level in the Apical corpus which overlaps with the maximal CK response [30, 31] (Fig 2B and 2C, and Fig A in S1 Appendix). In contrast, cells in the Basal corpus accumulate relatively lower amounts of WUS protein and exhibit a lower CK response [31]. Thus, to compare the influence of WUS and CK, we analyzed cell division plane orientation, cell shapes, and the direction of anisotropic cell expansion separately for the Apical corpus and Basal corpus.

#### WUSCHEL induces radial expansion of cells and cytokinin induces apical-basal expansion of cells

**Fig 9:**
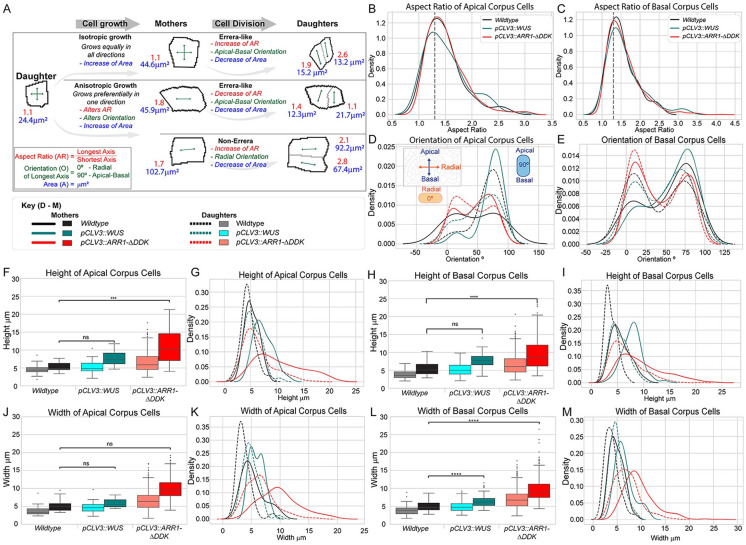
Ectopic misexpression of WUS and CK signaling influence the direction of anisotropic expansion of cells. (A) Patterns of anisotropic cell expansion have distinct outcomes on the aspect ratios and orientations of mother and new daughter cells. (B-C) Distributions of cell aspect ratios in wildtype (black), ectopic misexpression of WUS (teal), and ectopic misexpression of CK (red) experiments. (D-E) Distributions of mother and daughter cell orientations in wildtype (black, grey), ectopic misexpression of WUS (teal, cyan) and ectopic misexpression of CK (red, salmon) experiments are shown separately for all cells with aspect ratios above 1.3. Cells were segregated into mother and daughter cells by area. Distributions of mother and daughter cell (F,G) heights and (H,I) widths are shown separately for wildtype and ectopic misexpression experiments. Additional analysis of L1 and loss of functions mutants included in S6 Fig.

Observations of epidermal cell divisions in previously reported time series suggest that due to the small size of SAM cells they need to expand before dividing along a certain axis [22, 42]. While the degree of anisotropy of an elongated mother cell reflects anisotropic expansion, the degree of anisotropy of recently divided daughter cells is initially determined by the previous cell division plane orientation rather than its own anisotropic expansion (Fig 9A). To account for these two differences, we analyzed the distributions of cell orientations separately for mother (large) and daughter (small) cells. In what follows, we heuristically identify “mother cells” as those with area greater than the average computed for a given meristem, and “daughter cells” to be those with area smaller than the meristem average (see S1 Appendix for details). Moreover, we defined “elongated cells” to be those cells with aspect ratio ≥ 1.3. This threshold was determined because the 2D anisotropic expansion direction of cells with aspect ratio ≥ 1.3 more closely matches the cells they are taken from in 3D compared to cells with more isotropic shapes (see S2 Appendix for details).

To understand how WUS influences patterns of cell expansion in the SAM, we next analyzed the aspect ratios, orientations, widths, and heights of cells in ectopic misexpression of WUS experiments (Fig 9). First, we found that the distribution of cell aspect ratios was not significantly different from that of wildtype SAMs in either the Apical corpus or Basal corpus (Fig 9B and 9C). Next, we found that the distribution of mother cell orientations in the Apical corpus was significantly different from wildtype (p-value = 5.0e-2) (Fig 9D). In particular, our analysis revealed that the majority of mother cells in ectopic misexpression of WUS experiments were oriented in the apical-basal direction (i.e. 90°), whereas mother cell orientations in wildtype SAMs were more equally distributed between the radial and apical-basal directions (Fig 9D). Taken at face value, these results suggest that WUS induces apical-basal expansion of cells. However, this result is inconsistent with our previous conclusion that WUS inhibits periclinal cell divisions (Fig 4A).

Namely, according to Errera’s rule, an increase in apical-basal expansion of cells would result in an increase in periclinal cell divisions. On the other hand, if WUS overrides shape or stress-based cues to promote anticlinal cell divisions, then a WUS-driven increase in apical-basal expansion of cells followed by a WUS-driven increase in anticlinal cell divisions would result in more “non-Errera” divisions causing highly anisotropic cell shapes compared to wildtype SAMs (Fig 9A, bottom row). Since we did not observe a significant difference in the distribution of cell aspect ratios in ectopic misexpression of WUS SAMs compared to wildtype (Fig 9B), then it cannot be the case that WUS induces apical-basal expansion of cells. In addition, we observed a similar phenomenon in the Basal corpus of CK over-expressing SAMs (Fig 9E). Namely, we found a dramatic increase in the number of mother cells that were oriented in the radial direction compared to wildtype. However, by similar reasoning as above, since we did not observe a significant difference in the distributions of cell aspect ratios between ectopic misexpression of CK SAMs and wildtype SAMs (Fig 9B and 9C), then it cannot be the case that CK induces radial expansion of cells.

Thus, to further investigate how WUS and CK influence the direction of anisotropic expansion of cells, we next analyzed changes in cell widths and heights upon misexpression of WUS and CK. First, in ectopic misexpression of WUS SAMs, we only found a slight increase in the average cell width of mother and daughter cells in the Apical corpus (Fig 9J). However, we also observed the appearance of a second peak in the distribution of mother cell widths in this region (Fig 9K). In addition, we found a significant increase in the average cell width of mother and daughter cells in the Basal corpus compared to wildtype SAMs (Fig 9L and 9M). These two observations indicate an increase in the number of cells undergoing anisotropic expansion in the radial direction upon misexpression of WUS. Next, we observed a more significant increase in the average cell height of mother and daughter cells in both the Apical and Basal corpus (Fig 9F and 9H). However, we also found that the distributions of mother cell heights appeared to be a “shifted-right copy” of the daughter cell distributions in both regions (Fig 9G and 9I). These data not only suggest that WUS induces radial expansion of cells, but they provide insight into our previous results showing the majority of mother cells in ectopic misexpression of WUS experiments were oriented in the apical-basal direction.

Namely, these results indicate that cells are undergoing slight growth occurring perpendicular (apical- basal) to the primary direction of anisotropic expansion (radial) that builds over generations leading to the observed changes in the distribution of mother cell orientations in this condition. Similar observations of changes in cell heights and widths upon misexpression of CK indicate that CK induces apical-basal expansion of cells (Fig 9F–9M). (Note that the observed changes in the distribution of mother cell widths compared to daughter cell widths in the Apical corpus of ectopic misexpression of CK experiments is most likely due to the interference of CK with WUS.) Moreover, while the nuance of this interaction is difficult to resolve experimentally, computational model simulations testing this hypothesized mechanism of anisotropic expansion reproduce the experimental results we presented in this section. Thus, this detailed analysis of cell aspect ratios, orientations, widths, and heights in ectopic misexpression experiments support our model predictions that WUS and CK regulate anisotropic cell expansion in the corpus.

## Discussion

This paper aims to further elucidate the structure-function relationship between the mechanisms driving SAM growth and proper stem cell regulation in plants. Through comparing experimental and model simulation results obtained under multiple perturbation conditions, we confirmed that 1) in the Apical corpus, WUS and CK only regulate anisotropic expansion of cells and cell division plane orientation is determined based on tensile stress on the cell wall and 2) in the Basal corpus, WUS and CK regulate both cell division plane orientation and anisotropic expansion. Moreover, experimental results confirm our model prediction that this layer-specific, combined chemical and mechanical mechanism can maintain proper SAM shape, layered structure, and the correct distribution of WUS within the tissue. Hence, the results of this paper provide an additional link between the roles of WUS, CK, and mechanical stress in regulating pattern and shape during SAM morphogenesis. Cell and tissue level outcomes resulting from the testing of four hypothesized mechanisms of regulation compared to experiments are summarized in Tables 1 and 2.

**Table 1:** Summary of cell-level features resulting from the testing of four hypothesized mechanisms of regulation compared to experiments. N/A*: Heights and widths were not computed for the CAE-E mechanism since it failed to reproduce the percent periclinal and aspect ratio observed in wildtype experiments. N/A**: The combined mechanism was only simulated for wildtype conditions. *: Since we observed slightly more periclinal divisions in the Basal corpus for this mechanism compared to wildtype SAMs, this could explain why the distribution of mother cell orientations in this region does not directly match experiments. **: While we found that the distributions of mother cell widths for all three mechanisms were significantly different from daughters (inconsistent with experiments), all three mechanisms exhibit similar behavior to experiments in that we see an increase in the number of mother mother cells whose widths are above the average. ***: While we found that the distribution of mother cell heights was significantly different from daughters (inconsistent with experiments), the shape of the distributions for mother and daughter cell heights are similar to experiments. The second peak of the mother cell distribution could be due to the slightly higher number of periclinal divisions in this region compared to experiments. ****: While we found that the distributions of mother cell heights were significantly different from daughters (inconsistent with experiments), both mechanisms exhibit similar behavior to experiments in that we see an increase in the number of mother mother cells whose heights are above the average. *****: While we found that the distributions of mother cell widths were significantly different from daughters (inconsistent with experiments), both mechanisms exhibit similar behavior to experiments in that we see an increase in the number of mother mother cells whose widths are above the average. ******: While we observe significantly more periclinal divisions compared to experiments, the significant increase in periclinal divisions from the Apical corpus to the Basal corpus matched experimental trends.

Multiscale Model Simulations	Feature	Wildtype		*pCLV3::WUS*		*pCLV3::ARR1 dDDK*	
		Apical corpus	Basal corpus	Apical corpus	Basal corpus	Apical corpus	Basal corpus
CAE-E	Percent Periclinal	Very High	Low	Very High	Match	Very High	Very Low
	Aspect Ratios	No Match	No Match	No Match	No Match	No Match	No Match
	Orientations	Match	Match	No Match	No Match	Match	Match
	Heights	N/A*	N/A*	N/A*	N/A*	N/A*	N/A*
	Widths						
	Tensile Stress	Highest	Highest	Highest	Highest	Highest	Highest
CAE-M	Percent Periclinal	Match	Low	Match	Slightly Lower	Slightly Lower	Very Low
	Aspect Ratios	Match	Match	Match	Match	Match	Match
	Orientations	Match	Match	No Match	No Match	No Match	No Match
	Heights	No Match	No Match	Match****	No Match	No Match	No Match
	Widths	No Match	Match**	No Match	No Match	No Match	Match*****
	Tensile Stress	Lowest	Intermediate	Lowest	Lowest	Lowest	Intermediate
CED	Percent Periclinal	Match	Match	Very High	Match	Very High	Match******
	Aspect Ratios	Match	Match	Match	Match	Match	Match
	Orientations	Match	Match	No Match	No Match	Match	No Match
	Heights	No Match	Slight Match	Match****	No Match	Match	Match
	Widths	No Match	Match**	No Match	No Match	Match	Match*****
	Tensile Stress	Lowest	Intermediate	Intermediate	Lowest	Intermediate	Lowest
Combined	Percent Periclinal	Match	Match	N/A**	N/A**	N/A**	N/A**
	Aspect Ratios	Match	Match				
	Orientations	Match	Match*				
	Heights	Match	Match***				
	Widths	Match	Match**				
	Tensile Stress	Lowest	Lowest				

**Table 2:** Summary of tissue-level features resulting from the testing of four hypothesized mechanisms of regulation compared to experiments.

Mechanism	Feature	Wildtype	*pCLV3::WUS*	*pCLV3::ARR1 dDDK*
CAE-E	Layered Organization	Worst	Low Deviation	Low Deviation
	SAM Size	Good Match	High Variance	Similar
	SAM Shape	Too Flat	Too Low	Too Low
	WUS Distribution	Very Low	N/A	N/A
	Stem CellMaintenance	Too High		
CAE-M	Layered Organization	Intermediate	Low Deviation	Low Deviation
	SAM Size	High Variation	Slightly Larger	Similar
	SAM Shape	High Variation	Too Low	Slightly Lower
	WUS Distribution	Very Low	N/A	N/A
	Stem Cell Maintenance	Too High		
CED	Layered Organization	Intermediate	Low Deviation	Low Deviation
	SAM Size	Slightly Larger	Slightly Larger	Similar
	SAM Shape	Too Flat	Too Low	Match
	WUS Distribution	Slightly Lower	N/A	N/A
	Stem CellMaintenance	Too High		
Combined	Layered Organization	Best	N/A	N/A
	SAM Size	Best Match		
	SAM Shape	Best Match		
	WUS Distribution	Best Match		
	Stem CellMaintenance	Best Match		

Obtained results also complement several recent studies linking mechanical stress on the cell wall to macroscopic behavior of plant tissues [10, 43–45]. Our work provides further mechanistic insight into how stress on individual cell walls could regulate cell division plane orientation in the corpus. Namely, we found that model simulations assuming cell division plane orientation based on local patterns of tensile stress on the cell wall closely matched experimental data, while simulations assuming cell division plane orientation based on cell shape (i.e. Errera’s rule) did not. This is profound because it suggests that tensile stress caused by growth heterogeneity and other local interactions supersedes cell shape in controlling cortical microtubule orientation which plays a crucial role in cell wall deposition [10, 43, 46, 47].

While our knowledge of exactly how cells sense and interpret mechanical forces prior to cell division remains unclear, it has been demonstrated that microtubules directing microfibrils impact placement of the preprophase band (PPB) (a microtubule and microfilament structure that marks the cell division plane before mitosis) [46–48]. In addition, coordination of cell division among neighboring cells both within and across clonally distinct layers could be mediated by mechanical cues [22]. Although directly measuring mechanical stress in the internal cell layers of the SAM remains experimentally difficult, the quantitative image analysis of microtubule dynamics has been used to indirectly infer stress patterns in the distal portion of the SAM [10, 49]. Thus, similar quantitative approaches could provide a way to verify stress distributions from our computational model predictions to better understand how cells communicate via mechanical cues to regulate cell division plane orientation.

Our results also suggest possible feedback between tissue shape/structure and genetic regulators (i.e. regulators controlling cell division rate, cell wall organization, and cell division plane orientation) in determining organ function. Namely, we found that each hypothesized mechanism resulted in a different distribution of WUS within the SAM (Fig 8K and 8L). This suggests that the emergent tissue structure maintained by cell division plane orientations impacts how chemical signals spread throughout a tissue. For example, the non-dividing walls separating the L2 and L3 cell layers are expected to be limited in the number of primary plasmodesmata (PD) that develop during *de novo* cell wall synthesis, and that could limit apical movement of WUSCHEL. Along these same lines, periclinal cell divisions promoted by cytokinin signaling in the corpus not only result in the development of multiple cell layers, but they are also expected to increase the number of primary PD along the root-to-shoot axis that could play a key role in facilitating proper WUS diffusion in the SAM. While our understanding of the PD distribution and density in SAMs is limited, earlier experiments have demonstrated the prevalence of symplasmic domains which suggest the regulation of PD distribution in individual SAM layers [50–52]. In addition, other studies have shown that increasing the size of the WUS protein or decreasing aperture of the PD, blocks movement of WUS into the outer cell layers [29, 53, 54]. Thus, the modeling approach described in this paper will be extended to quantitatively link patterns of cell division plane orientation to regulation of PDs and WUS diffusion in *A. thaliana* SAMs.

In addition to the structural organization of the SAM, the WUS gradient is also regulated by positional signals such as CK, *CLAVATA3*-mediated receptor kinase signaling, and CLE40 [30, 31, 33]. These chemical signals act on WUSCHEL synthesis, subcellular partitioning, and degradation to establish the concentration gradient with robustness for regulating stem cell homeostasis. It could be the case that this biochemical signaling network is affected by mechanical cues or regulates structural components controlling cell behaviors that lead to proper SAM growth and stem cell maintenance. While the cellular basis of tissue homeostasis has been studied extensively, including in animal systems [55–60], the relationship between signaling pathways and mechanical cues requires further elucidation. Thus, quantitative studies utilizing experiments and multiscale computational models coupling cell mechanics and signal transduction have the potential to reveal important insights about the interplay between biochemical and mechanical processes regulating tissue growth and homeostasis.

We are working on overcoming several limitations of the current model by coupling our mechanical submodel of SAM growth with a dynamic signaling submodel in time, and calibrating the coupled model using dynamic experimental data. In particular, tracking individual cells in the corpus over time, and extracting dynamic data about changes in their size, shape, anisotropic expansion patterns, and division plane orientation in response to different mechanical and chemical signals is currently very challenging. However, insights from the current study focusing on understanding how the steady-state distributions of biochemical signaling molecules regulate cell division and tissue growth represent substantial work toward development of a mechanochemical coupled model as a future direction. In addition to developing a coupled model, extending our current 2D model to 3D is important for further testing of the hypothesized mechanisms we presented in this paper, especially concerning the unique mechanical properties of plant cells. While we have developed a 3D subcellular element (SCE) model of cell in the SAM (see S3 Appendix for details), running 3D computational model simulations is prohibitively computationally costly at this time even on very large and powerful computer clusters. Thus, parallelization on GPUs is necessary for speeding up simulations in order to run 3D computational model simulations with the same detailed representation of cells. An additional challenge for development of a 3D model is extracting and analyzing the 3D data we need from experiments to calibrate our 3D model, including tracking and analyzing cells in 3D reconstructed images.

## Materials and methods

### Model description

To study the interplay between chemical regulators and mechanical stresses in directing underlying cell behaviors and maintaining SAM structure and shape, we developed a detailed, multiscale, 2D computational model and calibrated it using experimental data. The model uses the subcellular element (SCE) computational framework to simulate a two-dimensional (2D) longitudinal section of the central region (as depicted in Fig 2A) of a growing SAM (see Fig 5, S1, S2 and S3 Videos for typical simulation output, and S3 Appendix for details on model initial and boundary conditions). The SCE modeling approach is a well-established, coarse-grained simulation framework for determining the impact of local biophysical and biochemical processes on emergent cell and tissue scale properties of growing or deforming multicellular tissues [61–72].

**Fig 10:**
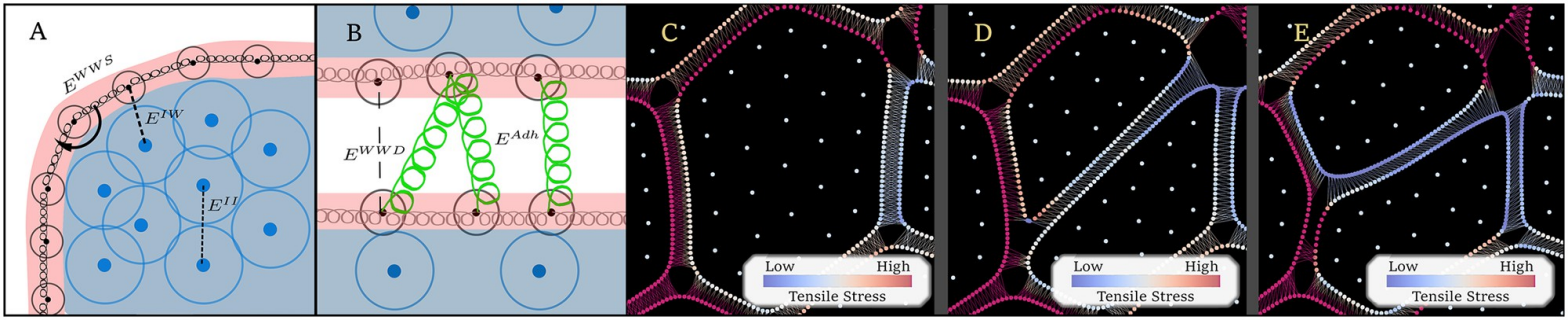
Two-dimensional multi-scale model of SAM growth and maintenance. Simulated cells are represented by two collections of nodes: cell wall nodes (solid black dots in the red region in A and B) and internal nodes (solid dark blue dots in the blue region in A and B). (A) Adjacent wall nodes of the same cell are shown interacting via linear and rotational springs given by *E*^*WW*^ that represent mechanical stiffness and extensibility of the primary cell wall. Pairs of internal nodes and internal and cell wall nodes of the same cell are shown interacting via Morse potential functions given by *E*^*II*^, *E*^*IW*^ that represent cell turgor-pressure. (B) Wall nodes of neighboring cells may form adhesion partners and interact via a linear spring potential given by *E*^*Adh*^ that represents the adhesive properties of the middle lamella. Wall nodes of adjacent cells enforce cell-cell volume exclusion via Morse potentials given by *E*^*WWD*^. The division process in the model is demonstrated in C-E, with the cell on display dividing in response to in-plane tensile stress. The heat map shown in C-E represents in-plane tensile stress tensile stress on each node where warmer colors represent nodes under higher tensile stress, and cooler colors represent nodes under lower tensile stress. (C) A simulated cell nearing mitotic phase. (D) A pair of simulated cells shortly after division. Cytoplasm nodes are redistributed within the daughter cells and adhesion partners of each wall node are updated. (E) The same cells as in D after an elastic relaxation phase occurs.

The novel, 2D, multiscale, SCE model described in this paper represents cells using two types of nodes- internal/cytoplasmic and external/cell wall nodes- that interact via different potential functions. Such biologically calibrated interactions between nodes simulate mechanical properties of plant cell walls facilitating novel predictions of how cell wall mechanics can help regulate the direction of anisotropic expansion of cells, and cell division plane orientation (see Figs 1 and 10, and S3 Appendix for details). Furthermore, an important distinction between our previous modeling approach [61] and the computational model presented in this paper, is the introduction and detailed testing of novel hypothesized mechanisms regulating cell division plane orientation and the modification of cell wall properties leading to anisotropic cell surface expansion (see section “Dependence of cell growth direction polarization and direction of anisotropic expansion on competing signals” and S3 Appendix for details). Both of these processes are thought to play an important role in emergent cell and tissue level properties of the SAM. In what follows, we provide a detailed description of the development, calibration, and implementation of SCE submodel components at distinct scales and how they are coupled to run multiscale simulations of SAM growth.

### Submodel of mechanical properties of cells and cell-cell interactions

Individual cells are modeled as possessing a heterogeneous collection of wall nodes and internal nodes (shown here in Fig 10A and 10B) which interact via potentials as in Banwarth-Kuhn et al. [61]. In particular, each cell *i* has *N*_*i*_ wall nodes $W_{i}^{j}$ (for *j* = 1, …*N*_*i*_) and *M*_*i*_ internal nodes $I_{i}^{j}$ (for *j* = 1, …, *M*_*i*_). The potential functions *E* in Eqs 1 and 2 represent specific biological features of plant cells (described in further detail below) and are used in the model to calculate the displacement of each internal or cell wall node at each time step based on their interactions with neighboring nodes. The Langevin equations of motion used in the model are as follows:

$$\begin{array}{cc} \eta_{i}\frac{d}{dt}W_{i}^{j}= & -\sum_{k=1}^{M_{i}}\nabla E^{IW}\left(W_{i}^{j},I_{i}^{k}\right)-\nabla E^{WWS}\left(W_{i}^{j},W_{i}^{j\pm 1}\right)\\ \; & -\sum_{\text{cells}\;l}\sum_{k=1}^{N_{l}}\nabla E^{WWD}\left(W_{i}^{j},W_{l}^{k}\right)-\sum_{\begin{array}{c} \text{adhesion}\\ \text{neighbors}\;\text{of}\;\text{cell}\;k \end{array}}\nabla E^{Adh}\left(W_{i}^{j},W^{k}\right) \end{array}$$ (1)

$$\begin{array}{c} \eta_{i}\frac{d}{dt}I_{i}^{j}=-\sum_{k=1}^{M_{i}}\nabla E^{II}\left(I_{i}^{k},I_{i}^{j}\right)-\sum_{k=1}^{N_{i}}\nabla E^{IW}\left(W_{i}^{k},I_{i}^{j}\right), \end{array}$$ (2)

where *η*_*i*_ is a cell’s damping coefficient. The Morse potential functions *E*^*II*^ and *E*^*IW*^ together represent coarse-grained cytoplasmic forces and resulting turgor-pressure of cells. The Morse potential function *E*^*WWD*^ represents volume exclusion of neighboring cells. Pairwise linear spring interactions (*E*^*Adh*^) between cell wall nodes of adjacent cells function as a coarse-grained model for cross-linking of pectin molecules in the middle lamella. The potential function *E*^*WWS*^ governs interactions between cell wall nodes of the same cell and is used to represent mechanical stiffness and extensibility of the primary cell wall. These functions comprise both linear and rotational spring potentials, as well as Morse potentials, given by:

$$\begin{array}{c} \begin{array}{cc} E^{WWS}\left(W_{i}^{j},W_{i}^{j+1}\right) & =\underset{\text{Linear}\;\text{spring}\;\text{potential}}{\underset{︸}{\frac{1}{2}k_{\text{lin}}[{\left(|W_{i}^{j+1}-W_{i}^{j}|-\ell \right)}^{2}+{\left(|W_{i}^{j-1}-W_{i}^{j}|-\ell \right)}^{2}]}}\\ & +\underset{\text{Rotational}\;\text{Spring}\;\text{Potential}}{\underset{︸}{\frac{1}{2}k_{\text{bend}}{\left(\theta -\theta_{\text{eq}}\right)}^{2}}}, \end{array}\\ \begin{array}{cc} E^{IW}\left(W_{i}^{k},I_{i}^{j}\right)= & \underset{\text{Turgor}\;\text{Pressure}}{\underset{︸}{\left[U^{IW}\text{exp}\left(-\frac{\left|W_{i}^{k}-I_{i}^{j}\right|}{\xi^{IW}}\right)-W^{IW}\text{exp}(-\frac{\left|W_{i}^{k}-I_{i}^{j}\right|}{\gamma^{IW}})\right]}},\\ E^{WWD}\left(W_{i}^{j},W_{l}^{k}\right)= & \underset{\text{Volume}\;\text{Exclusion}}{\underset{︸}{\left[U^{WWD}\text{exp}(-\frac{\left|W_{i}^{j}-W_{l}^{k}\right|}{\xi^{WWD}})-W^{WWD}\text{exp}(-\frac{\left|W_{i}^{j}-W_{l}^{k}\right|}{\gamma^{WWD}})\right]}},\\ E^{Adh}\left(W_{i}^{j},W^{k}\right)= & \underset{\text{Cell-Cell}\;\text{Adhesion}}{\underset{︸}{\frac{k_{\text{Adh}}}{2}{\left(|W_{i}^{j}-W^{k}|-\ell^{\text{Adh}}\right)}^{2}}},\\ E^{II}\left(I_{i}^{k},I_{i}^{j}\right)= & \underset{\text{Cytoplasm}\;\text{Pressure}}{\underset{︸}{\left[U^{II}\text{exp}(-\frac{\left|I_{i}^{k}-I_{i}^{j}\right|}{\xi^{II}})-W^{II}\text{exp}(-\frac{\left|I_{i}^{k}-I_{i}^{j}\right|}{\gamma^{II}})\right]}} \end{array} \end{array}$$ (3)

where $W_{i}^{j\pm 1}$ denotes the positions of the nodes adjacent to node $W_{i}^{j}$, and *θ* is the angle formed by $W_{i}^{j}$ and $W_{i}^{j\pm 1}$. Ranges for the parameters *k*_bend_, *k*_lin_, *θ*_eq_, and *ℓ* were calibrated based on the modulus of elasticity, sizes, and shapes of cells measured in experiments (see section “Dependence of cell growth direction polarization and direction of anisotropic expansion on competing signals” and S3 Appendix for details on sensitivity analysis and calibration of these parameters). The Morse parameters ξ*, γ*, U* and W* were chosen based on coarse graining resolution and discussed in [61, 73]. In simulations, the exact values used for these parameters are dynamic and change in response to a probability distribution function parameterized by the amount of signal (WUS and CK) present in the cell at a given time (see the section “Dependence of cell growth direction polarization and direction of anisotropic expansion on competing signals” for details). The explicit parameter values for all other potential functions were calibrated in previous work [61, 73].

Each simulation represents 40 hours of tissue growth. The Euler numerical scheme was used for solving Eqs 1 and 2. The time step Δ*t* was chosen to be 0.4 seconds to maintain stability of the numerical scheme. Growth rates and cell cycle lengths are discussed in more detail in the section “Chemical signal distribution submodel controls growth of cells”.

### Chemical signal distribution submodel controls growth of cells

The concentrations of WUSCHEL ([WUS]) and cytokinin ([CK]) for individual cells are assigned using the experimentally-calibrated exponential functions given in Eqs 4 and 5. While multiple feedback loops are known to regulate WUS and CK at both the transcriptional and protein levels, their net effect has been shown to result in steady-state distributions of WUS and CK in the SAM [31, 34]. Thus, Eqs 4 and 5 in the model were calibrated based on the steady-state distributions of WUS and CK measured from experimental images as in [61], by neglecting the mechanism underlying the establishment of such gradients. These functions describe the concentrations of WUS and CK as being distributed with radial symmetry about a dynamically determined point, the *signal center*, which represents the middle of the signals’ expression domains. Values of [CK] in L1 and L2 are maintained at 0, since in wildtype SAMs these cells do not show CK responsiveness, which is likely due to the limitation of the CK reception system [35, 36]. Concentrations for WUS, as well as concentrations for CK in the corpus, are independently calculated using the expressions:

$$\begin{array}{c} \left[\text{WUS}\right]={\left[\text{WUS}\right]}_{0}\text{exp}(-\mu_{\text{WUS}}\left(r_{\text{WUS}}\cdot \alpha_{\text{WUS}}\right)); \end{array}$$ (4)

$$\begin{array}{c} \left[\text{CK}\right]={\left[\text{CK}\right]}_{0}\text{exp}(-\mu_{\text{CK}}\left(r_{\text{CK}}\cdot \alpha_{\text{CK}}\right)), \end{array}$$ (5)

where *r*_WUS_ and *r*_CK_ are the distance from the centroid of each cell to the WUS and CK signal centers, respectively.

**Fig 11:**
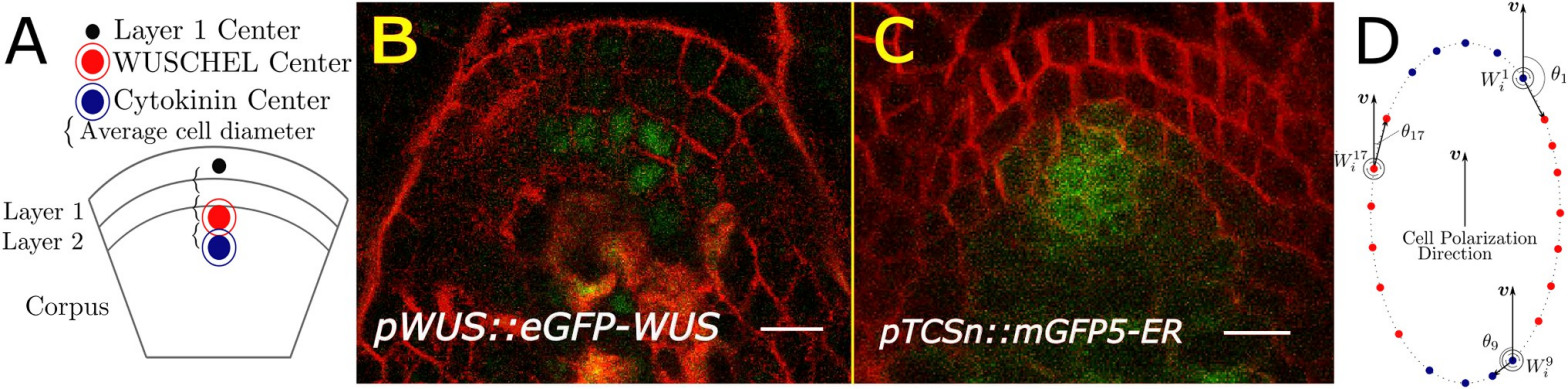
Chemical signaling submodel of the SAM. (A) Schematic of model signal expression domains of WUSCHEL and cytokinin. (B-C) Wildtype SAM longitudinal section image. Cell walls (red) outline the dome-shape of the SAM; reporters (green) indicate presence of the WUSCHEL protein (B) and cytokinin reporter (C). (B-C) Contrast was manually increased in the red channels for visibility. Scale bars are 10*μm*. (D) Cell growth direction polarization ***v*** is dependent upon signaling. Wall nodes of a model cell polarized in direction ***v*** are illustrated. The angle *θ*_*j*_ between ***v*** and the vector connecting $W_{i}^{j}$ to $W_{i}^{j+1}$ are used to partition the wall nodes into sides (red) and ends (blue).

In experiments, the WUS and CK expression domains are located below the distal portion of the SAM, heuristically described as being located two and three times the length of the average cell diameter beneath the distal portion of the SAM, respectively (Fig 11B and 11C). We established the signal center locations for WUS and CK similarly in simulations, setting them as two and three times the average diameter of the tunica cells, respectively, directly below the centroid of the central L1 cell (Fig 11A). The signal centers are updated dynamically throughout the course of simulations to ensure that the center of the WUS and CK expression domains in simulations maintain their position relative to the growing distal portion of the SAM, as observed in experiments. Parameters *μ*_WUS_, *μ*_CK_, [WUS]_0_, and [CK]_0_ were fitted to experimental wildtype data in [61] for *α*_WUS_ = 1 and *α*_CK_ = 1. Parameters [WUS]_0_, [CK]_0_, *α*_WUS_, and *α*_CK_ were then perturbed to simulate under/over-expression or ectopic distribution of signals corresponding to experimental mutant conditions.

In simulations, individual cell cycle lengths (the amount of time between two successive divisions of an individual cell) are dynamically assigned based on the current WUS concentration of individual cells as in [61]. The cell cycle length is chosen using a normal distribution parameterized by [WUS], and calibrated using experimental data (data from [22]; calibration method as in [61]). New cytoplasm nodes are added linearly in time until the cell divides upon having 30 internal nodes. The process of division is detailed in the section “Dependence of cell division plane orientation on chemical regulators and in-plane tensile stress”.

### Dependence of cell growth direction polarization and direction of anisotropic expansion on competing signals

Cells in the SAM experience turgor-pressure driven expansion, and the orientation and level of alignment of microfibril bundles within the cell wall can promote preferential cell expansion along one axis of a cell [6, 41, 74]. We refer to this phenomenon as *growth direction polarization*, which we capture by making the stiffness and equilibrium angle of the cell wall’s rotational springs heterogeneous across wall nodes in a cell (Fig 11D). Cells that are growing on the simulation boundary grow isotropically, so the rotational spring parameters are chosen to be uniform. All other cells do so anisotropically, detailed below.

Model signals of WUS and CK compete to direct cell growth direction polarization, where CK promotes growth direction polarization in the apical-basal direction and WUS promotes growth direction polarization in the radial direction. Each cell’s growth direction polarization is signal-determined as in the section “Stochastic antagonistic signaling between WUSCHEL and cytokinin”. We then set the stiffness and equilibrium angle of the rotational spring for *E*^*WWS*^ (see Eq 3) heterogeneously across the cell’s wall nodes, representing microfibril-bound wall nodes (sides) and freely growing wall nodes (ends) as in Fig 11. The side nodes (red) have a much stiffer rotational spring, *k*_bend^high^_, whose equilibrium angle is set to *π* (flat), while the ends (blue) have a much looser rotational spring, *k*_bend^low^_, whose equilibrium angle set to prefer a circular arrangement—i.e. $\frac{\pi (N_{i}-2)}{N_{i}}$ where *N*_*i*_ is the number of wall nodes possessed by cell *i*. The explicit values of the rotational spring stiffness coefficients,*k*_bend^high^_ and *k*_bend^low^_, were determined via a sensitivity analysis optimizing the range of observed areas and aspect ratios of individual cells in single-cell simulations to the range of areas and aspect ratios measured in wildtype experiments [73] (see Table A in S3 Appendix for exact parameter values and S3 Appendix for more details on sensitivity analysis studies).

### Dependence of cell division plane orientation on chemical regulators and in-plane tensile stress

When model cells complete a mitotic cycle (i.e. reach 30 internal nodes), they divide. Layer 1 and 2 cells are always prescribed to divide with a plane normal to the SAM surface as observed in experiments. To do this, we defined anticlinal division planes to be perpendicular to the line segment connecting its in-layer neighbors’ cell centers. In the corpus, each simulated meristem follows one of four hypothesized mechanisms determining the position of the planes of division. The individual mechanisms are described in the following subsections.

Following the choice of division plane, two daughter cells are formed. These daughter cells are created out of the mother’s original wall nodes and redistributed cytoplasm nodes, as well as new cell wall nodes along the division plane which represent the new cell wall and middle lamella. These newly formed cells are adhered to one another (Fig 10C–10E).

#### Cell division based on Errera’s Rule (CAE-E mechanism)

Errera’s rule states that the division plane chosen for a cell should result in the shortest possible cell wall [75]. This method selects the plane passing through the pair of nodes that minimizes the distance $∥W_{i}^{j}-W_{i}^{k}∥$ while evenly dividing the cell’s area $\left(0.9\leq \frac{A_{1}}{A_{2}}\leq 1.11\right)$.

#### Cell division based on in-plane tensile stress (CAE-M mechanism)

The in-plane tensile stress *S* on a cell wall element $W_{i}^{j}$ is calculated as the average mechanical force exerted on it by its neighbors in the tangential direction, as follows:

$$\begin{array}{ll} S_{i,j}= & \frac{1}{2}\cdot ∥{\text{Proj}}_{\tau }\left(\frac{W_{i}^{j}-W_{i}^{j-1}}{∥W_{i}^{j}-W_{i}^{j-1}∥}k_{\text{lin}}\left(∥W_{i}^{j}-W_{i}^{j-1}∥-l\right)\right)∥\\ & +\frac{1}{2}\cdot ∥{\text{Proj}}_{{}_{\tau }}\left(\frac{W_{i}^{j}-W_{i}^{j+1}}{∥W_{i}^{j}-W_{i}^{j+1}∥}k_{\text{lin}}\left(∥W_{i}^{j}-W_{i}^{j+1}∥-l\right)\right)∥, \end{array}$$ (6)

where denote ***τ*** as a unit vector tangent to the surface of the cell, $W_{i}^{j}$ is the node’s location as 2D a vector, and *ℓ* and *k*_lin_ are the equilibrium length and stiffness of the linear spring part of *E*^*WWS*^ (see Eq (3). We then calculate $S(W_{i}^{j})$ for all wall nodes in the cell and select location of the maximally stressed wall node as one point on the division plane. The second node defining the division plane is chosen so that the cell area divides evenly $\left(0.9\leq \frac{A_{1}}{A_{2}}\leq 1.11\right)$. We observe in simulations that the distribution of in-plane tensile stress along the cell wall is smooth, and the largest variation in stress is near cell wall junctions. In most cases, the node experiencing maximal tensile stress is indicative of a local region of high tensile stress around that node, demonstrating that a single-node based selection method is sufficient for the CAE-M mechanism. While our model is in 2D, an analogous approach could be used for a 3D model as described in S3 Appendix.

#### Cell division based on chemical signals (CED mechanism)

Under this mechanism, cells under relatively high levels of CK are more likely to divide periclinally, and cells under the influence of WUS will likely divide anticlinally. The anticlinal versus periclinal behavior of the cell is determined by the probability distribution described in the section “Stochastic antagonistic signaling between WUSCHEL and cytokinin”. If the cell divides periclinally, the cell area divides evenly ($0.9\leq \frac{A_{1}}{A_{2}}\leq 1.11$) with a horizontal plane; otherwise it divides anticlinally—that is, evenly divided and with a vertical plane.

#### Layer-specific combined chemical signaling and mechanical mechanism

In this mechanism, cells whose lineage are traced back to the third and fourth layers of the initial conditions divide according to the CAE-M mechanism, as in the section “Cell division based on in-plane tensile stress (CAE-M mechanism)”. Cells in layers below that divide according to the CED mechanism, as in the section “Cell division based on chemical signals (CED mechanism)”.

### Stochastic antagonistic signaling between WUSCHEL and cytokinin

Two novel hypothesized mechanisms we tested using our model include 1) whether WUS and CK can regulate cell growth direction polarization and 2) how WUS and CK regulate cell division plane orientation. Thus, in our model, cell behavior is influenced by WUS to expand and divide anticlinally and by CK to expand and divide periclinally. However, we assume that every cell responds to these competing signals with some uncertainty—abstractly representing any heterogeneity in the cells’ sensitivity to the signal. To represent this, we use the relative signal—the ratio of WUS signal in a cell to the CK signal in a cell—to parameterize the probability distribution used to determine cells’ behavior.

More specifically, noise in the competition between WUS and CK is modeled by considering the ratio λ = [CK]/[WUS] as a parameter for a probability mass function:

$$\begin{array}{cc} \text{Prob}(\text{Cell}\;\text{follows}\;\text{periclinal}\;\text{behavior}) & =\frac{1}{1+{\left(\frac{K_{\text{Hill}}}{\lambda }\right)}^{N_{\text{Hill}}}};\\ \text{Prob}(\text{Cell}\;\text{follows}\;\text{anticlinal}\;\text{behavior}) & =1-[\frac{1}{1+{\left(\frac{K_{\text{Hill}}}{\lambda }\right)}^{N_{\text{Hill}}}}]. \end{array}$$

*K*_Hill_ was calculated by imposing that the midpoint between the signaling domains (section Chemical signal distribution submodel controls growth of cells) have equally probable anticlinal and periclinal behavior, and as such *K*_Hill_ ≔ [CK]/[WUS]|_***x***=Signal Center_. The value of *N*_Hill_ was fitted experimentally to experimentally observed anticlinal-periclinal division ratios under wildtype conditions.

### Plant growth and genotypes

Plants were grown on 0.5X MS media in plates at 25°C under continuous light for 7–8 days. The null mutants in this study: WUS null mutant—*wus1–1* [32] and cytokinin triple receptor mutant—*cre1–12; ahk2–2; ahk3–3* [37] have been previously described. Transgenic plants containing fluorescent reporters for the WUS protein *pWUS::eGFP-WUS* [29] and cytokinin signaling reporter *pTCS::mGFP-ER* [35, 36] have also been previously described. A two component system, consisting of a LhG4 transcription factor driven from *CLAVATA3 promoter* constitutively activating the 6xOP promoter, and a dexamethasone (Dex) inducible rat glucocorticoid receptor (GR) were used for ectopic misexpression of the WUS protein *pCLV3::LhG4;6xOP::eGFP-WUS-GR* [24] and CK signaling *pCLV3::LhG4;6xOP::ARR1*-Δ*DDK-GR* [31]. For induction of ectopic expression, seedlings were transferred to 0.5X MS plates containing 10 *μ*M Dex (Sigma) for 48 hours.

### Imaging

Seedlings were embedded in molten 4% agarose and then chilled in an ice bath. Longitudinal hand-dissections, using polished blades (FEATHER), were done through the seedling and the supporting agarose. Samples were then submerged in plasma membrane stain, FM4–64, for 10 minutes and imaged with a 40x objective lens on the Zeiss LSM 880 and Leica SP5 confocal microscopes. For 3D analysis of cells, the inflorescence meristems were stained with FM4–64 and confocal cross sections were obtained by acquiring z-stack on the Zeoss LSM 880. FM4–64 staining was activated with 561 nm—Zeiss or 543 nm—Leica SP5 and collected with Airyscan detector—Zeiss or collection window 600–650 nm—Leica SP5. eGFP and mGFP were activated with 488 nm and collected between 525–550 nm. Metrics to calculate cell and tissue values from imaging analysis and simulations are detailed in S1 Appendix.

Additional materials and methods used in this study are described in S1, S2 and S3 Appendices.

### Statistical analysis

Statistical tests were implemented using the statannot package in python [76]. Statistical analyses of the data (p-values and type of test) are presented in the Results section.

## Supporting information

S1 Appendix Image segmentation, quantification and analysis. **Fig A:** **Multiple levels of SAM organization.** (A) An annotated longitudinal section through a wildtype shoot apical meristem (SAM) and organ primordia. Clonal layers (B) and distinct functional zones (C) of the SAM. (D) Annotated cell walls from inferred daughters cells after division. Anticlinal cell divisions are shown in yellow and periclinal cell divisions are shown in cyan. (E) Overlay representing the nuclear WUS protein distribution (green). (F) Overlay representing TCS reporter of cytokinin signaling (purple). (G) Four features used to determine cell division plane orientation. Segmentation output of wildtype (H),ectopic misexpression of CK [pCLV3::LhG4; 6xOP::ARR1-ΔDDK-GR] (I), and ectopic misexpression of WUS [pCLV3::LhG4; 6xOP::eGFP-WUS-GR] (J) experimental SAMs. Line segments inside cells are provided to help visual each individual cell’s aspect ratio and orientation. The length of a cell’s line segment is proportional to its aspect ratio- where cell’s with aspect ratio = 1 have line segments with length 0. The directional vector of each line segment represents the orientation of the longest axis of the encompassing cell. Orange denotes cells that are classified as small cells and blue denotes cells that are classified as large cells (see S1 Appendix Section C for details on analyses comparing large and small cell characteristics).
(PDF)

S2 Appendix Justification of 2D experimental methods. **Fig A:** **Verification of 2D section analysis as a proxy for 3D.** (A-B) The principal direction of elongation for both 2D sections (A) and 3D cells (B) are shown in blue. The direction of apical-basal axis (taken to be the Z axis) is shown in red, and the angle between them are the azimuthal angles, which we use to classify cells as anticlinally or periclinally expanded. The 3D cell and section are taken from a z-stack image of a wildtype SAM. The units of the axes are in microns. The origin point of both the 2D and 3D axes are arbitrary. (C) The difference between the 2D and 3D cell azimuthal angles taken from 3D cells and their longitudinal section is shown on the vertical axis. The horizontal axis is the aspect ratio of the cell sections, with larger values representing more dramatically elongated cell sections. The threshold chosen for aspect ratio ≥1.3 is indicated by the vertical red line, and the tolerance of 15° is shown as a horizontal line. Cell sections analyzed in the 2D experimental analysis are those cells to the right of the vertical line. The aspect ratio threshold of 1.3 was chosen to include a significant portion of data, while ensuring cell sections were elongated enough to well-represent the behavior of the 3D cell.
(PDF)

S3 AppendixExtended model description.**Table A:** **Main parameter values for simulations.** Parameters that varied in *in-silica* experiments. **Table B:** **Initial conditions for simulations.** Variables that control the initial configuration of the system. **Fig A:** **Initial conditions and equilibrium state.** (A) 50 model cells and their initial adhesion connections between neighboring cell wall nodes are shown in the initial layout used for each simulation. (B) An example of an equilibrium state achieved after stage one of simulations. Note that in the equilibrium state, each cell has been stochastically assigned a direction of cell growth polarization, leading to anisotropically expanded cells at mechanical equilibrium. (A-B) Cells on the left and right sides of the simulated tissue domain are the boundary cells which do not divide in any stage of the simulation, but otherwise obey the same rules as other cells. Cells in the bottom most layer are considered part of the stem, and have a much higher damping to provide a foundation for the expansion of the SAM. The heat map shows the distribution of in-plane tensile stress as calculated in Eq (6) in the main text. **Fig B:** **Aspect ratio and orientation of cells.** Nodes $W_{i}^{s^{1}}$ and $W_{i}^{s^{2}}$ (highlighted nodes) are chosen to evenly divide the cell area with minimal segment length. The perpendicular bisector is formed and nodes nearest are taken to be the long axis $W_{i}^{l^{1}}$ and $W_{i}^{l^{2}}$ (highlighted nodes). The growth direction angle *θ* of cell *i* is the positive acute angle between the horizontal and the long axis. Aspect ratio is also calculated from the lengths of the long and short axes. Orientation is measured in the same way as for experimental images described above. Image was rendered via simulation output, and the heat map shows tensile stress calculated by node as in Eq (6) in the main text. **Fig C:** **Perturbation Analysis of Equilibrium State.** Perturbation analysis results of adding small, random displacements to initial cell locations on the mechanical equilibrium state of the tissue—i.e. after representing *t* = 15 minutes of growth, as discussed in section D. The top left and top center panels demonstrate both the impact of cell center displacement on global curvature and how that impact may be attributed to the fitness of a circle to the surface. The top right panel shows that the apical surface length of the equilibrium SAM is only impacted via a negligible increase in variation of the total apical surface length. The bottom panels show all cell-level measurements of non-boundary cell orientation, aspect ratio, and areas from 100 simulations of increasing centroid noise amplitude. These panels exhibit independence of cell geometry and orientation from random cell displacement—suggesting that the initial tiling’s precise spatial arrangement does not play a role in the equilibrium distribution of cells. **Fig D:** **Sensitivity Analysis of Equilibrium State.** Tissue scale (top) and cell-scale (bottom) measurements taken from simulated SAMs after 15 minutes of simulated growth were represented as in section D. Values of *k*^lin^ (left 6 panels) and *η*_Boundary_ (right 6 panels) were independently varied to 100 values around their default. (Top) all tissue-scale impacts on boundary dynamics were negligible, with the exception of the reduction of apical surface length with a fourfold increase of $k_{\text{Boundary}}^{\text{lin}}$. However, this is attributable to the shrinkage of the boundary cells with high *k*^lin^ on the boundary which, upon observation, appear to pull the SAM surface flatter. This difference is less than half of a micron, and so we conclude that the overall impact of boundary cell properties on tissue-scale measurements is negligible. (Bottom) Local sensitivity analysis shows no impact of passive boundary mechanical properties on equilibrium cell shape. **Fig E:** **CAE-M division plane mechanism in 3D model.** (A) Diagram of a 3D model cell is shown in gray and depicts the cross-section of the division plane predicted by the CAE-M mechanism in blue. The vector *v*_1_ (green) is used to determine the cell division plane by finding the plane that includes both *v*_1_ and *N*_Max_, and that divides the cell volume approximately in half. (B) Diagram of the calculation of *v*_Max_, the maximal stress direction in $T_{N_{\text{Max}}}$ within a neighborhood of *N*_Max_, and the resulting vector *v*_1_ in $T_{N_{\text{Max}}}$ orthogonal to *v*_Max_. Vectors indicating stresses acting on *N*_Max_ as a result of nearby cell wall nodes are indicated in black, and their projection onto the tangent plane $T_{N_{\text{Max}}}$ is shown in red. **Fig F:** **CAE-M division plane in 3D cell and corresponding 2D model plane.** (A) 3D SAM (black lines) with cell (blue) intersected by the 2D longitudinal plane (green) our model simulates. (B) 3D cell (blue) intersected by the 2D model plane (green). The front face of the cell shows the out-of-plane node with maximal stress and maximal stress direction (solid yellow arrow) found using the 3D version of our CAE-M mechanism. The right face of the cell shows the in-plane node with maximal stress and maximal stress direction (dashed yellow arrow) found using the 2D version of our CAE-M mechanism based on in-plane tensile stresses. (C) 3D cell intersected by 2D model plane (green) and the division plane predicted by the 3D CAE-M mechanism (brown). The line segment predicted by the 2D CAE-M mechanism lies at the intersection of the 3D division plane and the 2D model plane (solid white line).(PDF)

S1 Fig Cytokinin signaling increases periclinal cell divisions in Basal Corpus. Consecutive periclinal division lead to the formation of strips in wildtype (black) and ectopic misexpression of cytokinin (red). 4-cell strips are caused by three sequential periclinal divisions and 10-cell strips are caused by nine sequential periclinal division. Significance was determined by student T-test for ectopic misexpression of CK signaling compared to wildtype. Asterisks indicate significance at the following levels ****p < 0.0001.
(PDF)

S2 Fig Comparison of cell orientations, heights, and widths between experimental wildtype SAMs and wildtype simulations for all four hypothesized mechanisms. The distributions of cell orientations for mother (solid lines) and daughter cells (dashed lines) in the (A) apical corpus and (B) basal corpus. The distributions of (C) cell heights and (D) cell widths in the apical corpus. The distribution of (E) cell heights and (F) cell widths in the basal corpus. In all Fig, experimental data (black), CAE-E (blue), CAE-M (gold), and CED(green). Significance was determined by Levene’s test.
(PDF)

S3 Fig Ectopic misexpression of CK influences the direction of anisotropic cell expansion. The distributions of (A) cell aspect ratios and (B-D) orientations in the Apical and Basal corpus from ectopic misexpression of CK experiments [pCLV3::LhG4; 6xOP;ARR1- Δ DDK-GR] and simulations comparing three hypothesized mechanisms. (A,B) The distributions for all cells in experimental (black), CAE-E (blue), CAE-M (gold), and CED (green). The distributions of cell orientations for (C) mother cells (solid lines) and (D) daughter cells (dashed lines) were segregated based on cell size and independently graphed. The distributions of (E-F) cell heights and (G-H) cell widths in the Apical corpus for the ectopic misexpression of CK condition. The distributions of (I-J) cell heights and (K-L) cell widths in the Basal corpus for the ectopic misexpression of CK condition. (M) Amount of deviation from a single-cell layer in the epidermal L1 and L2 cell layers for experimental SAMs (black), CAE-E simulations (blue), CAE-M simulations (gold), and CED simulations (green) in the ectopic misexpression of CK condition. (N) The ratio of SAM width to dome height for experimental SAMs (black), CAE-E simulations (blue), CAE-M simulations (gold), and CED simulations (green) in the ectopic misexpression of CK condition. (O) Global curvature of the SAM surface for experimental SAMs (black), CAE-E simulations (blue), CAE-M simulations (gold), and CED simulations (green) in the ectopic misexpression of CK condition. See S3 Appendix for detailed description of all metrics used in this Figure.
(PDF)

S4 Fig Ectopic misexpression of WUS influences the direction of anisotropic cell expansion. The distribution of (A) cell aspect ratios and (B-D) orientations in the apical corpus and basal corpus from ectopic misexpression of WUS in experiments [pCLV3::LhG4; 6xOP;eGFP-WUS-GR] and simulations comparing three hypothesized mechanisms. (A,B) The distribution for all cells in experimental (black), CAE-E (blue), CAE-M (gold), and CED (green). The distribution of cell orientations for (C) mother cells (solid lines) and (D) daughter cells (dashed lines) were segregated based on cell size and independently graphed. The distributions of (E-F) cell heights and (G-H) cell widths in the apical corpus of cells in ectopic misexpression of WUS condition. The distributions of (I-J) cell heights and (K-L) cell widths in the basal corpus of cells in ectopic misexpression of WUS condition. (M) Amount of deviation from a single-cell layer in the epidermal L1 and L2 cell layers for experimental SAMs (black), CAE-E simulations (blue), CAE-M simulations (gold), and CED simulations (green) in the ectopic misexpression of WUS condition. (N) The ratio of SAM width to dome height for experimental SAMs (black), CAE-E simulations (blue), CAE-M simulations (gold), and CED simulations (green) in the ectopic misexpression of WUS condition. (O) Global curvature of the SAM surface for experimental SAMs (black), CAE-E simulations (blue), CAE-M simulations (gold), and CED simulations (green) in the ectopic misexpression of WUS condition. See S3 Appendix for detailed description of all metrics used in this Figure.
(PDF)

S5 Fig Time evolution of simulated cell orientations and aspect ratios by condition and division plane mechanism. The distributions of cell orientations (Left) and aspect ratios (Right) at various time points computed directly from simulations, organized by cell division plane mechanisms and perturbation condition. The distributions of cell orientations and aspect ratios were obtained for the combined mechanism only in the wildtype simulations.
(PDF)

S6 Fig WUS and CK misexpression and loss of function mutants influence the direction of anisotropic expansion of cells. (A) Aspect ratio and (B) orientation of L1 and L2 cells in from wildtype, ectopic misexpression of WUS [pCLV3::LhG4; 6xOP::eGFP-WUS-GR], and ectopic misexpression of CK [pCLV3::LhG4; 6xOP::ARR1-ΔDDK-GR] experimental SAMs. (C-F) Cell layer specific aspect ratio and orientation of cells from wildtype, *wus1* mutants [*wus1–1*], and cytokinin triple receptor mutants[*cre1;ahk2;ahk3*]. Cell height (G) and width (H) of L1 and L2 cell layers for each experimental condition. Significance was determined by t-test for each experimental condition compared to wildtype. Asterisks indicate significance at the following levels:****p < 0.0001.
(PDF)

S1 Video Typical simulation output of 40 hours of growth for all four mechanisms (CAE-E, CAE-M, CED, and combined) in wildtype signaling conditions. (MP4)

S2 Video Typical simulation output of 40 hours of growth for three mechanisms (CAE-E, CAE-M, and CED) in ectopic misexpression of CK signaling conditions. (MP4)

S3 Video Typical simulation output of 40 hours of growth for three mechanisms (CAE-E, CAE-M, and CED) in ectopic misexpression of WUS signaling conditions. (MP4)
