# Supplementary material for: Combined computational modeling and experimental analysis integrating chemical and mechanical signals suggests possible mechanism of shoot meristem maintenance
Source: PLoS Comput Biol. 2022 Jun 21;18(6):e1010199. doi: 10.1371/journal.pcbi.1010199 (PMC9249181; doi:10.1371/journal.pcbi.1010199)
Supplement: S1 Appendix — Fig A: Multiple levels of SAM organization. (A) An annotated longitudinal section through a wildtype shoot apical meristem (SAM) and organ primordia. Clonal layers (B) and distinct functional zones (C) of the SAM. (D) Annotated cell walls from inferred daughters cells after division. Anticlinal cell divisions are shown in yellow and periclinal cell divisions are shown in cyan. (E) Overlay representing the nuclear WUS protein distribution (green). (F) Overlay representing TCS reporter of cytokinin signaling (purple). (G) Four features used to determine cell division plane orientation. Segmentation output of wildtype (H),ectopic misexpression of CK [pCLV3::LhG4; 6xOP::ARR1-ΔDDK-GR] (I), and ectopic misexpression of WUS [pCLV3::LhG4; 6xOP::eGFP-WUS-GR] (J) experimental SAMs. Line segments inside cells are provided to help visual each individual cell’s aspect ratio and orientation. The length of a cell’s line segment is proportional to its aspect ratio- where cell’s with aspect ratio = 1 have line segments with length 0. The directional vector of each line segment represents the orientation of the longest axis of the encompassing cell. Orange denotes cells that are classified as small cells and blue denotes cells that are classified as large cells (see S1 Appendix Section C for details on analyses comparing large and small cell characteristics). (PDF) [file pcbi.1010199.s001.pdf]

S1. Image segmentation, quantification and analysis

A. Image segmentation. Images were manually cropped using MATLAB to focus on the distal portion of the SAM. Each cropped micrograph was segmented in MATLAB by isolating the color channel containing stained plasma membrane and passing it through a 2-D Gaussian filter with a standard deviation of 2.5. An h-minima transformation was then applied, with the h value optimized to minimize the variance of cell areas in each tissue region. This was followed by a watershed transformation to segment the image and remove cells on the boundary of the image. All functions mentioned were part of MATLAB's Image Processing Toolbox.

B. Feature quantification. Segmentation resulted in a mask compatible with MATLAB's "regionprops" measurements, which included calculations of cell centroids, areas, major and minor axis lengths, and orientations (see Fig A, panels H, I, and J). We then manually annotated the layer identity of each cell as L1, L2, or corpus (see Fig A, panel B). The *depth* of each cell was calculated by measuring the distance from the centroid of the cell to the center of the L1 cell layer, where the center of the L1 cell layer was defined to be the mean of all centroids belonging to cells in the L1 cell layer. The *aspect ratio* of a cell was calculated to be the ratio of the longest axis to the shortest axis of the cell. To calculate the *width* of the SAM, the horizontal distance between the left-most and right-most cell centroids among all L1 cell centroids was calculated for each SAM image. To calculate the *dome height* of the SAM, the vertical distance between the lowest and highest cell centroids among all L1 cell centroids was calculated for each SAM image (see Fig 3F in the main text).

B.1. Definition and quantification of anticlinal and periclinal divisions. Classically, anticlines and periclines are used to quantify the patterning of division plane placement relative to the nearest tissue surface or sub-epidermal cell layers (1, 2). While developmentally relevant, such definitions present problems in the present study because these axes implicitly require placement of vertices in the corpus based on SAM shape which may require inconsistent or arbitrary heuristics that vary considerably between some of the more deformed mutant phenotypes (e.g. flat vs. enlarged meristems), ultimately leading to ambiguous definitions of anticlines and periclines for the present work. Since we are considering cells near the tunica in the central zone and for the above reasons, we define anticlinal and periclinal growth and division relative to the apical-basal axis in both simulations and experimental images. In the CZ, the conservative classifications made with our method would likely align well with classification of anticlines and periclines in the natural coordinate system as presented in (1, 2), provided that the center of the natural coordinate system was placed below the SAM. Moreover, we acknowledge that we are using this terminology as a metric for establishing phenotypic distinction between meristems, which is independent of the original developmental context behind the classic definition of anticlines and periclines.

Pairs of cells that descended from the division of a single precursor cell (i.e. sibling cells) were manually identified based on four characteristics: 1) small longitudinal section areas, 2) similar cell sizes, 3) straight flanking walls on the lateral edges of the cell pair and 4) one cell wall shared exclusively between the cell pair (see Fig A, panel G). To categorize division planes into anticlinal and periclinal, the apical-basal axis was used as an absolute reference for the vertical direction. This is because near the central zone (see Fig 2A in the main text), the curvature of the SAM is not pronounced enough to have significant disparity between the apical-basal direction and the direction normal to the surface of the SAM. With the apical-basal axis taken as vertical, we defined the *orientation* of the newly-placed cell wall as the angle that the plane makes with the radial (i.e. horizontal) axis. More specifically, since the data are two-dimensional, new cell walls appear as lines, and the orientation is the acute angle between that line and the horizontal axis. Anticlinal divisions are those divisions with orientation $\geq 60^\circ$ and periclinal divisions are those divisions with orientation $\leq 30^\circ$. More detail on the selection of these thresholds is given in S2 Appendix. Once a pair of sibling cells was identified, the orientation of the new cell wall between them was quantified as either *anticlinal* (perpendicular to the SAM surface) or *periclinal* (perpendicular to the apical-basal axis). The number of divisions of each type was counted for a minimum of 10 experimental samples for each signaling condition.

B.2. Quantification of the direction of anisotropic cell expansion. In this study, we define the *orientation* of an individual cell as the acute angle between the radial (i.e. horizontal) axis and the longest axis of the cell. This value is given in degrees, ranging from 0° to 90° . In this way, the orientation of a cell describes its direction of anisotropic expansion relative to the SAM tissue. For example, a cell with orientation equal to 0° is expanded primarily along the radial axis of the SAM and a cell with orientation equal to 90° is expanded primarily along the apical-basal axis of the SAM. We define cells as being *elongated* if the cells have aspect ratio > 1.3. This threshold was determined because elongated cell sections in 2D have their anisotropic expansion direction better-represent the cells they are taken from in 3D (see S2 Appendix for details).

B.3. Quantification of the levels and spatial patterns of chemical signaling. The WUSCHEL (WUS) levels of individual cells were measured using fluorescence intensity from isolated color channels reporting the WUS protein in experimental SAM images for wildtype condition as shown in Fig 11B in the main text. The WUS levels of individual cells and their corresponding cell depths were then analyzed and fit to an exponential function as in (3). This process was repeated for SAM images containing the cytokinin (CK) distribution, which was visualized similarly (see Fig 11C in the main text).

C. Large and small cell classification. We are using cell size as a proxy to classify cells as recently divided or about to divide. In order to get the volume of data to perform the analyses presented in this work, we relied on methods precluding the acquisition of time-series data. As such, using size as a proxy for the pre-mitotic or post-mitotic state of a cell was a methodological necessity. For analyses comparing large and small cell characteristics separately, we heuristically identify "large" cells as those

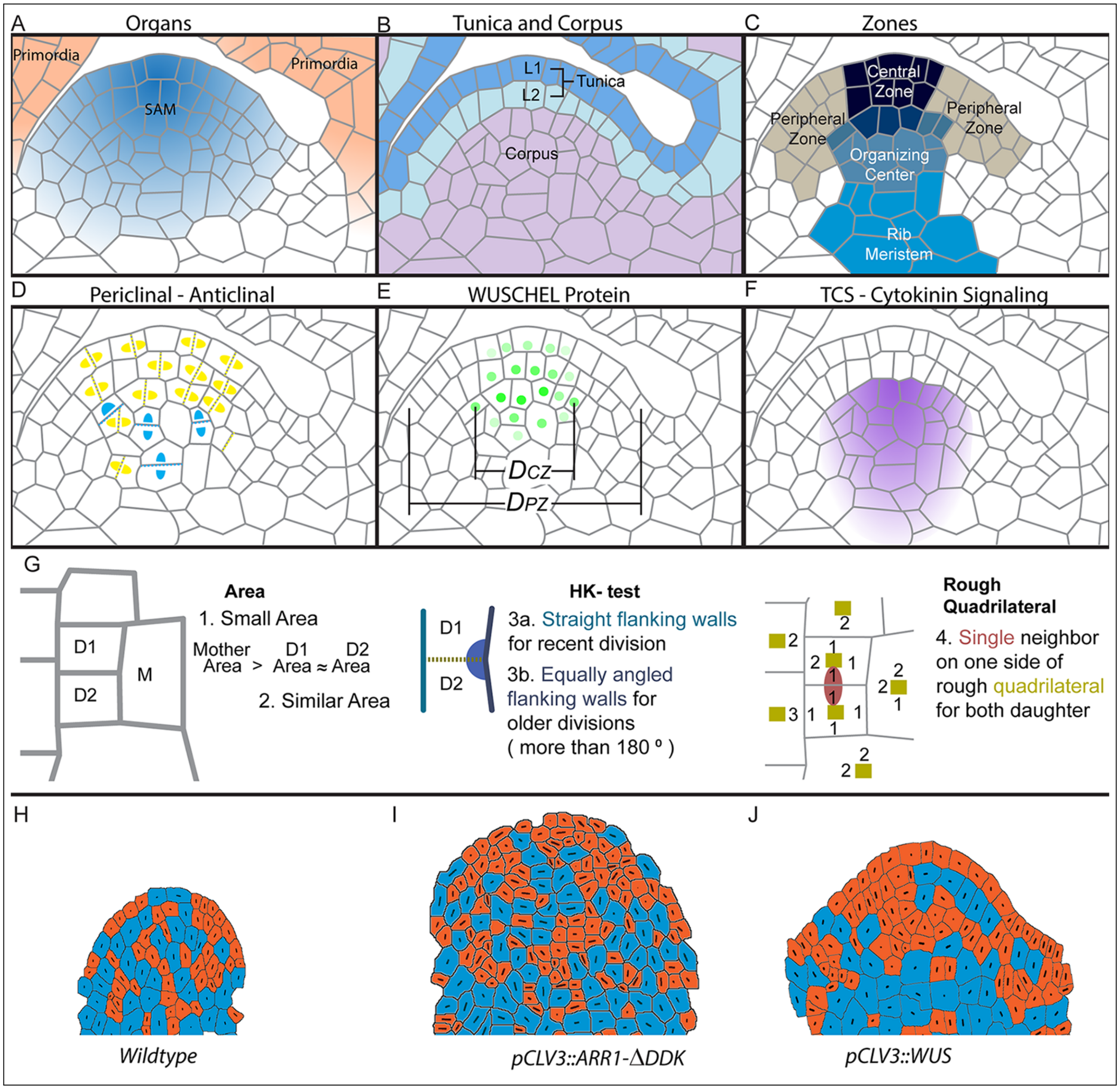

Fig. A. Multiple levels of SAM organization. (A) An annotated longitudinal section through a wildtype shoot apical meristem (SAM) and organ primordia. Clonal layers (B) and distinct functional zones (C) of the SAM. (D) Annotated cell walls from inferred daughters cells after division. Anticlinal cell divisions are shown in yellow and periclinal cell divisions are shown in cyan. (E) Overlay representing the nuclear WUS protein distribution (green). (F) Overlay representing TCS reporter of cytokinin signaling (purple). (G) Four features used to determine cell division plane orientation. Segmentation output of wildtype (H),ectopic misexpression of CK [pCLV3::LhG4; 6xOP::ARR1-ΔDDK-GR] (I), and ectopic misexpression of WUS [pCLV3::LhG4; 6xOP::eGFP-WUS-GR] (J) experimental SAMs. Line segments inside cells are provided to help visual each individual cell's aspect ratio and orientation. The length of a cell's line segment is proportional to its aspect ratio- where cell's with aspect ratio = 1 have line segments with length 0. The directional vector of each line segment represents the orientation of the longest axis of the encompassing cell. Orange denotes cells that are classified as small cells and blue denotes cells that are classified as large cells (see S1 Appendix Section C for details on analyses comparing large and small cell characteristics).

with a greater than average cell area within the SAM section the cell was taken from, and “small” cells to be those cells with lower than average cell area within the SAM section the cell was taken from.

References

1. D Kwiatkowska, Structural integration at the shoot apical meristem: models, measurements, and experiments. *Am. journal botany* **91**, 1277–1293 (2004).
2. Z Hejnowicz, J Karczewski, Modeling of meristematic growth of root apices in a natural coordinate system. *Am. J. Bot.* **80**, 309–315 (1993).
3. M Banwarth-Kuhn, et al., Cell-based model of the generation and maintenance of the shape and structure of the multilayered shoot apical meristem of arabidopsis thaliana. *Bull. Math. Biol.* **81**, 3245–3281 (2019).
