# Supplementary material for: Combined computational modeling and experimental analysis integrating chemical and mechanical signals suggests possible mechanism of shoot meristem maintenance
Source: PLoS Comput Biol. 2022 Jun 21;18(6):e1010199. doi: 10.1371/journal.pcbi.1010199 (PMC9249181; doi:10.1371/journal.pcbi.1010199)
Supplement: S2 Appendix — Fig A: Verification of 2D section analysis as a proxy for 3D. (A-B) The principal direction of elongation for both 2D sections (A) and 3D cells (B) are shown in blue. The direction of apical-basal axis (taken to be the Z axis) is shown in red, and the angle between them are the azimuthal angles, which we use to classify cells as anticlinally or periclinally expanded. The 3D cell and section are taken from a z-stack image of a wildtype SAM. The units of the axes are in microns. The origin point of both the 2D and 3D axes are arbitrary. (C) The difference between the 2D and 3D cell azimuthal angles taken from 3D cells and their longitudinal section is shown on the vertical axis. The horizontal axis is the aspect ratio of the cell sections, with larger values representing more dramatically elongated cell sections. The threshold chosen for aspect ratio ≥1.3 is indicated by the vertical red line, and the tolerance of 15° is shown as a horizontal line. Cell sections analyzed in the 2D experimental analysis are those cells to the right of the vertical line. The aspect ratio threshold of 1.3 was chosen to include a significant portion of data, while ensuring cell sections were elongated enough to well-represent the behavior of the 3D cell. (PDF) [file pcbi.1010199.s002.pdf]

S2. Justification of 2D experimental methods

A. Feature quantification across neighboring longitudinal section images. To determine whether feature quantification of two-dimensional, longitudinal section images was robust to the choice of median longitudinal axis, the distributions of cell aspect ratios and orientations were compared between neighboring longitudinal section images for 9 wildtype experimental SAMs. Five to ten parallel, longitudinal section images were taken from each meristem spaced apart by 1.26 microns. Cell aspect ratios and orientations were quantified for each image as described in S1 Appendix. Distributions of cell aspect ratios and orientations were compared across all longitudinal section images for each meristem using the one-way analysis of variance (ANOVA). It was determined that the distributions of cell-scale features were not significantly different between longitudinal section images.

B. Comparison of 3D vs. 2D experimental data. The use of a 2D model suggests that for a more accurate direct experimental comparison, 2D experimental data should be used. However, we would like to confirm that we may draw meaningful conclusions about an intrinsically 3D system from 2D data. Specifically, the orientation of the cell section was predicted to be highly correlated with the orientation of the cell in 3D among elongated cell sections - i.e. cell sections with large aspect ratios.

To test this prediction, 2D sections and 3D reconstructions of multiple cells were compared to one another. Two 3D Z-Stacks of wildtype inflorescent SAMs were imaged at 0.33 micron intervals producing a voxel size of 0.13 x 0.13 x 0.33 microns. These 3D images were segmented by using a deep neural network that was trained to predict, for each cell in 3D, the distance between each pixel from the boundary of the cell containing that pixel. The prediction includes the coefficients required in order to spatially fit the cell's volume in 3D to spherical harmonics, and the error in this process was minimized by training the network on a published, hand-segmented training dataset of multiple SAMs(1). This segmentation method was performed in python from the pytorch library, and this method was detailed by Eschweiler et al. in (2).

This produced two 3D reconstructions of the SAM with 49.5 and 42.9 μm depth, respectively. Using these reconstructed 3D SAM images, we took multiple longitudinal sections of the reconstructed SAM using open-access MATLAB packages to triangulate the reconstructed SAM's cells and intersect them with the cell boundaries. Each plane passed through the apex of the SAM, and was parallel to the apical-basal axis. Five such planes - each different from one another by a rotation about the apical-basal axis - were used to generate 2D cross-sections taken from the reconstruction. Each 2D section image was then analyzed using MATLAB packages (including regionprops) as before to obtain 2D features such as orientation, aspect ratio, and area. Each cell whose section was analyzed also had its 3D properties analyzed in MATLAB's regionprops3 package to extract information such as the 3D orientation of each cell.

To confirm that the orientation direction of elongated cells in 2D well-represented the orientation direction of cells in 3D in their ability to categorize cells as anticlinal or periclinal, we calculated the angle between the orientation vectors and the apical-basal axis (i.e. the *azimuthal angle*, which is equivalent to 90° minus the orientation of a cell) both in 2D as well as 3D for each cell section (see Fig A, panels A and B). In the 3D setting, we define cells that are anisotropically expanded periclinally as those with azimuthal angle $\leq 45^\circ$ and anticlinally as those with azimuthal angle $\geq 45^\circ$.

It was found that for aspect ratio greater than 1.3, 80% of cell sections had their 2D azimuthal angle within 15 degrees of the azimuthal angle measured from the 3D cell (see Fig A, panel C). Categorizations of cell sections as anticlinally or periclinally expanded were made by placing the *section* azimuthal angle between 0° and 30° for periclinal, and 60° to 90° for anticlinal. This analysis ensures that with 80% probability, anisotropically expanded cells counted as anticlinal or periclinal by 2D section observation only give accurate representations of the cells' anisotropically expanded in 3D. That is to say, by considering cell sections with aspect ratio ≥ 1.3, we ensure:

$$\text{Prob(2D Section azimuthal angle } \leq 30^\circ \text{ and 3D azimuthal angle } \geq 45^\circ) \leq 20\%, \text{ and}$$
$$\text{Prob(2D Section azimuthal angle } \geq 60^\circ \text{ and 3D azimuthal angle } \leq 45^\circ) \leq 20\%.$$

This is equivalently stated as

$$\text{Prob(2D Section is classified as periclinal and 3D cell is classified as anticlinal)} \leq 20\%, \text{ and}$$
$$\text{Prob(2D Section is classified as anticlinal and 3D cell is classified as periclinal)} \leq 20\%.$$

Moreover, it should be noted that by observing the data (visualized in Fig A, panel C), larger threshold values, e.g. aspect ratio 1.6, would improve confidence from 80% to 90%, however it would decrease the amount of experimental data points available. Therefore the threshold of aspect ratio ≥ 1.3 was chosen. Furthermore, by using the ranges of $0^\circ - 30^\circ$ and $0^\circ - 45^\circ$ to classify cells as periclinally elongated in 2D and 3D respectively, and similarly using $60^\circ - 90^\circ$ and $45^\circ - 90^\circ$ to classify cells as anticlinally expanded, 91% of 2D cell sections identified as anticlinal or periclinal agreed with their 3D classification. These considerations indicate that, at least among elongated longitudinal cell sections, the orientation of a cell from a 2D section provides a good estimation of the true 3D cell orientation.

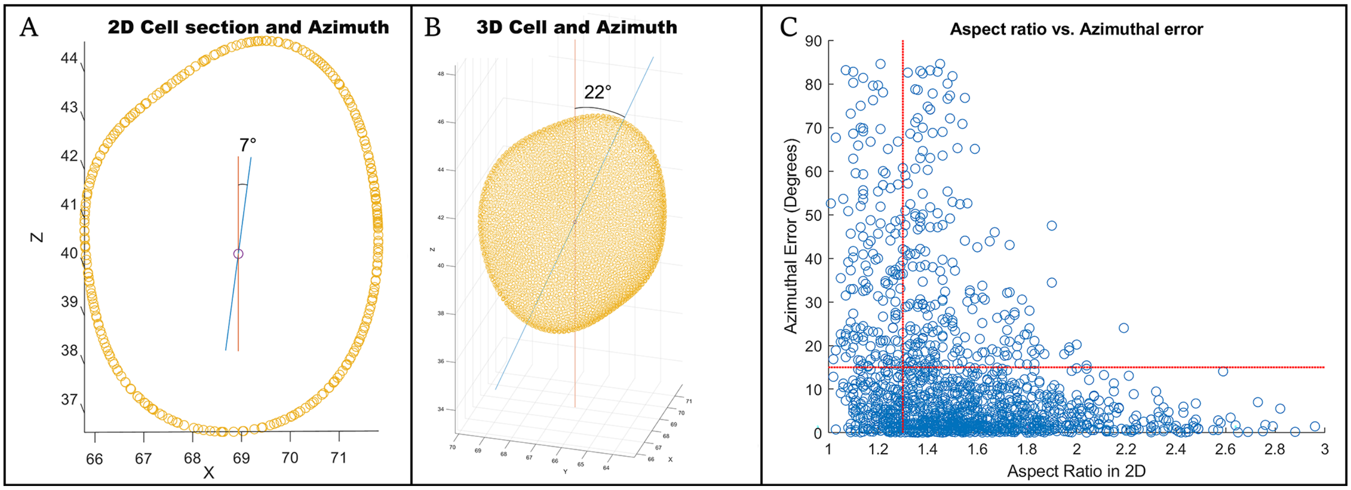

Fig. A. Verification of 2D section analysis as a proxy for 3D. (A-B) The principal direction of elongation for both 2D sections (A) and 3D cells (B) are shown in blue. The direction of apical-basal axis (taken to be the Z axis) is shown in red, and the angle between them are the azimuthal angles, which we use to classify cells as anticlinally or periclinally expanded. The 3D cell and section are taken from a z-stack image of a wildtype SAM. The units of the axes are in microns. The origin point of both the 2D and 3D axes are arbitrary. (C) The difference between the 2D and 3D cell azimuthal angles taken from 3D cells and their longitudinal section is shown on the vertical axis. The horizontal axis is the aspect ratio of the cell sections, with larger values representing more dramatically elongated cell sections. The threshold chosen for aspect ratio ≥ 1.3 is indicated by the vertical red line, and the tolerance of 15° is shown as a horizontal line. Cell sections analyzed in the 2D experimental analysis are those cells to the right of the vertical line. The aspect ratio threshold of 1.3 was chosen to include a significant portion of data, while ensuring cell sections were elongated enough to well-represent the behavior of the 3D cell.

References

1. L Willis, et al., Cell size and growth regulation in the arabidopsis thaliana apical stem cell niche. *Proc. Natl. Acad. Sci.* **113**, E8238–E8246 (2016).
2. D Eschweiler, M Rethwisch, S Koppers, J Stegmaier, Spherical harmonics for shape-constrained 3d cell segmentation in *2021 IEEE 18th International Symposium on Biomedical Imaging (ISBI)*. pp. 792–796 (2021).
