# Supplementary material for: Combined computational modeling and experimental analysis integrating chemical and mechanical signals suggests possible mechanism of shoot meristem maintenance
Source: PLoS Comput Biol. 2022 Jun 21;18(6):e1010199. doi: 10.1371/journal.pcbi.1010199 (PMC9249181; doi:10.1371/journal.pcbi.1010199)
Supplement: S3 Appendix — Table A: Main parameter values for simulations. Parameters that varied in in-silica experiments. Table B: Initial conditions for simulations. Variables that control the initial configuration of the system. Fig A: Initial conditions and equilibrium state. (A) 50 model cells and their initial adhesion connections between neighboring cell wall nodes are shown in the initial layout used for each simulation. (B) An example of an equilibrium state achieved after stage one of simulations. Note that in the equilibrium state, each cell has been stochastically assigned a direction of cell growth polarization, leading to anisotropically expanded cells at mechanical equilibrium. (A-B) Cells on the left and right sides of the simulated tissue domain are the boundary cells which do not divide in any stage of the simulation, but otherwise obey the same rules as other cells. Cells in the bottom most layer are considered part of the stem, and have a much higher damping to provide a foundation for the expansion of the SAM. The heat map shows the distribution of in-plane tensile stress as calculated in Eq (6) in the main text. Fig B: Aspect ratio and orientation of cells. Nodes Wis1 and Wis2 (highlighted nodes) are chosen to evenly divide the cell area with minimal segment length. The perpendicular bisector is formed and nodes nearest are taken to be the long axis Wil1 and Wil2 (highlighted nodes). The growth direction angle θ of cell i is the positive acute angle between the horizontal and the long axis. Aspect ratio is also calculated from the lengths of the long and short axes. Orientation is measured in the same way as for experimental images described above. Image was rendered via simulation output, and the heat map shows tensile stress calculated by node as in Eq (6) in the main text. Fig C: Perturbation Analysis of Equilibrium State. Perturbation analysis results of adding small, random displacements to initial cell locations on the mechanical equilibrium state of the tissue—i. [file pcbi.1010199.s003.pdf]

S3. Extended model description

A. Model Parameters. A Table provides parameter values used in simulations. The μ_* and $[*]_0$ values for WUS and CK were experimentally fitted in (1). Values for α_X were defined to be 1 for wildtype, and varied by factors that replicated observed [CK] : [WUS] ratios. Ranges of model parameters controlling mechanical stiffness and extensibility of the primary cell wall (k_{lin}, ℓ, $k_{\text{bend}}^{\text{loose}}$, and $k_{\text{bend}}^{\text{stiff}}$) were calibrated in (1, 2) so that the modulus of elasticity (E) of cells lies within the biological range. Additionally, global sensitivity analysis was used in (2) to quantify the impact of each of these parameters on the area and aspect ratio of cells in simulations. Then the parameters with the most impact on these two cell-level features were fit based on the area and aspect-ratio of cells measures in experiments. There are many other parameters of the model, e.g. parameters controlling the strength of cell-cell adhesion and for coarse-graining parameters, which have been described previously in (1, 2).

Table A. Main parameter values for simulations. Parameters that varied in *in-silica* experiments.

Parameter	Value	Interpretation	Chosen by
μ_{WUS}	0.01573 a.u.	Exponential fitting parameter	Calibrated in (1)
μ_{CK}	0.01637 a.u.	Exponential fitting parameter	Calibrated in (1)
α_{WUS}	0.4-1.5 a.u.	Ectopic misexpression factor	See Methods
α_{CK}	0.4-1.5 a.u.	Ectopic misexpression factor	See Methods
$[\text{WUS}]_0$	84.6 a.u.	Maximum WUSCHEL intensity	Calibrated in (1)
$[\text{CK}]_0$	110 a.u.	Maximum cytokinin intensity	Calibrated in (1)
N_{Hill}	10 a.u.	Steepness of stochastic threshold. Deterministic as $N_{\text{Hill}} \to \infty$.	See Methods
K_{Hill}	1.3 a.u.	[CK]/[WUS] yielding even CK-WUS competition	See Methods
ℓ	0.9 μm	Coarse graining parameter; linear spring equilibrium length	Calibrated in (1)
k_{lin}	280 $\frac{nN}{\mu m}$	Cell wall extensibility	Calibrated in (1, 2)
$k_{\text{bend}}^{\text{loose}}$	4.543 $\frac{nN}{\text{rad}}$	Loose wall bending spring constant - See Methods	Calibrated in (1, 2)
$k_{\text{bend}}^{\text{stiff}}$	13.5 $\frac{nN}{\text{rad}}$	Stiff wall bending spring constant - See Methods	Calibrated in (1, 2)
θ_{eq}	π or $\frac{\pi(N_i-2)}{N_i}$ rad	Equilibrium rotational spring angle	See Methods

B. Initial and Boundary Conditions. The initial "wedge" shape of the tissue (see Fig A, panel A) and the initial number of cells in each layer used as the geometry input for model simulations were calibrated based on previously published results by our group (3). These results provide average measurements of the central zone of the SAM, indicating that the number of central zone cells in Layer 1 is between 7-9 cells and decreases in a wedge-like shape across the next 3 layers. Additionally, we verified using this data that variability in both the "wedge" shape of the tissue and the number of cells in each layer is low. Our model simulates a 2D longitudinal section of the central region of the SAM (as depicted in Fig 2 A in the main text) that includes the central zone together with the organizing center and rib meristem. So, we start each simulation with 10 cells each in the first layer, where the outermost cell on each side acts as a boundary cell (see below), and include 4 additional corpus layers encompassing the organizing center and rib meristem (see Fig A, panel A and panel C of Fig A in S1 Appendix). The staggered configuration of cells is used to minimize the number of four-cell rosettes (as very few are observed in experiments (4)). To ensure that our precise tiling did not meaningfully impact simulations, we performed a perturbation analysis which is described in section D below.

To model the tissue boundary, a single layer of cells at the bottom and sides of this configuration do not divide during the simulation to act as a boundary, representing the neighboring cells around the CZ. Cells beneath the simulated region are assumed to be differentiating and expanding, and act as a barrier to prevent downward expansion of the SAM. We capture this by giving the bottom-most layer of cells a higher damping coefficient ($\eta_i = 10$ for cells i in the deepest layer) compared to all other cells ($\eta_i = 1$). Each cell is initialized as being partway through its cell cycle when the simulation begins, so we choose the initial number of cytoplasm nodes M_i to be uniformly random between 15 (recently divided daughter cell) and 29 (pre-mitotic, fully grown mother cell). B Table provides values for the initial conditions used in simulations along with a description of how each variable was calibrated.

Table B. Initial conditions for simulations. Variables that control the initial configuration of the system.

Variable	Value or Pattern	Calibration
Number of (Non-boundary) L1 cells	8 Cells	Experimentally quantified in (6)
Initial Tissue Shape	"Wedge", Experimentally quantified in (6)	
Tiling of L2-L7 cells	Staggered/Hexagonal grid	Minimizing 4-way junctions
Initial signaling distribution	Spatial distribution as in Methods	Calibrated from experiments
Cell Growth Direction	Stochastically chosen as in Methods	Calibrated from experiments
**Parameters controlling boundary cell behavior**		
$k_{\text{Boundary}}^{\text{lin}}$	$560.3272 \frac{nN}{\mu m}$	Local SA, see section S2D
η_{Stem}	10	Local SA, see section S2D
Centroid Noise Amplitude A	0	Local SA, see section S2D

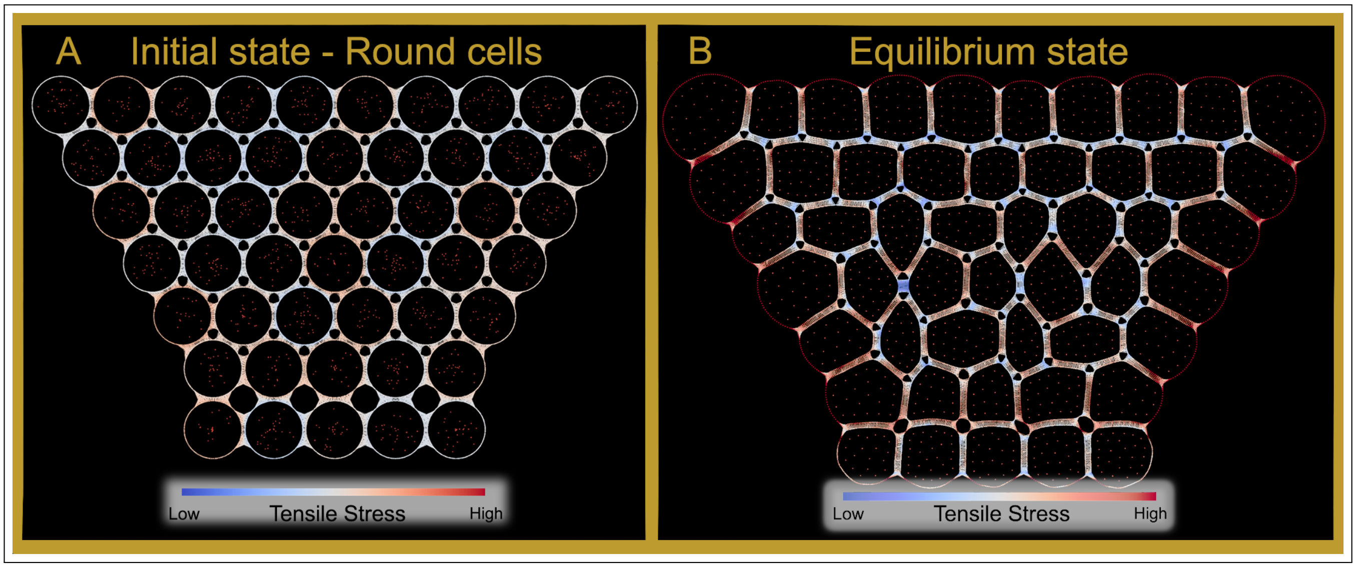

Fig. A. Initial conditions and equilibrium state. (A) 50 model cells and their initial adhesion connections between neighboring cell wall nodes are shown in the initial layout used for each simulation. (B) An example of an equilibrium state achieved after stage one of simulations. Note that in the equilibrium state, each cell has been stochastically assigned a direction of cell growth polarization, leading to anisotropically expanded cells at mechanical equilibrium. (A-B) Cells on the left and right sides of the simulated tissue domain are the boundary cells which do not divide in any stage of the simulation, but otherwise obey the same rules as other cells. Cells in the bottom most layer are considered part of the stem, and have a much higher damping to provide a foundation for the expansion of the SAM. The heat map shows the distribution of in-plane tensile stress as calculated in equation (6) in the main text.

All simulations consist of two stages. The first stage starts with circular cells arranged in the experimentally observed "wedge" shape representing the central region of the SAM (see Fig A, panel A). Then the simulation is run without cell growth and division to achieve an equilibrium of the system resulting in specific cell shapes based on experiments as well as a distribution of stresses for each cell (see Fig A, panel B). The second stage makes up the main simulation and starts with the equilibrium state achieved in stage one. In this way, the distribution of cell shapes used as initial conditions for the main simulation are representative of experimentally observed cell shape distributions. During the second stage, cells are allowed to grow, divide, and dynamically change their cell growth direction polarization in time as described in the Model Description section in the main text.

C. Metrics. Simulation outputs consist of collections of nodes' locations, WUS and CK signal concentration, cell identity within the tissue (i.e. layer), and information tracking the plane of divisions. What follows are the metrics applied to these data for comparison of the model to experimental data. Where applicable, experimental data were manually annotated to have directly analogous metadata (e.g. manual identification of the layers of each cell) in order to calculate these metrics using experimental images as well.

C.1. Anticlinal and Periclinal Division Metrics. Division data was recorded for all cells during simulations, including both the orientation of the chosen division plane and the layer the division occurred in. We report only on the layer 3 and below divisions, since L1 and L2 orient divisions via a local rule that always yields anticlinal divisions.

For each cell division j in layers 3 and below in a simulated meristem, consider $\{\Theta_j\}$ to be the set of acute angles the division planes made relative to the horizontal, so each $\Theta_j \in [0, 90°]$. We subdivide this interval to categorize our divisions as we would in experimentation:

$$\underbrace{[0°, 30°]}_{\substack{\text{Periclinal}\\ \text{Division}\\ \text{Range}}};\quad \underbrace{(30°, 60°)}_{\substack{\text{Diagonal}\\ \text{Division}\\ \text{Range}}};\quad \underbrace{[60°, 90°]}_{\substack{\text{Anticlinal}\\ \text{Division}\\ \text{Range}}}.$$

Letting A be the number of divisions whose Θ_j fall within the anticlinal division range and P be the number of divisions whose Θ_j that fall into the periclinal division range, the proportion of periclinal divisions for each cell layer was calculated as $P/(A + P)$. The partition of $[0, 90°]$ was chosen to match those used for experimental observations.

C.2. Aspect ratio and orientation of Cells. The aspect ratio and orientation of cells in all modeling results were computed directly from simulation data in the following manner. To determine the aspect ratio of a cell, we find the pair of cell wall nodes $W_i^{s^1}, W_i^{s^2}$ that define the short axis, i.e. the line segment with minimal segment distance that evenly divides the cell. The perpendicular bisector of the short axis is drawn to find the direction of the long axis of the cell (see Fig B). The wall nodes closest to the long axis on either side of the cell are taken to be the endpoints of the longest axis itself $W_i^{l^1}, W_i^{l^2}$. The aspect ratio is then determined as the ratio $\frac{\left\| W_i^{l^1} - W_i^{l^2} \right\|}{\left\| W_i^{s^1} - W_i^{s^2} \right\|}$. Cell orientation is measured using the long axis in the same way as

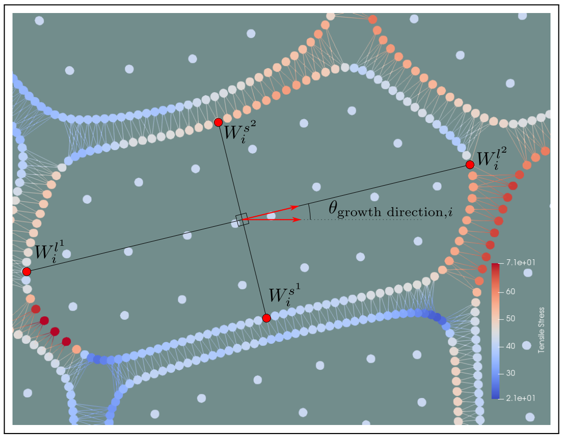

Fig. B. Aspect ratio and orientation of cells. Nodes $W_i^{s^1}$ and $W_i^{s^2}$ (highlighted nodes) are chosen to evenly divide the cell area with minimal segment length. The perpendicular bisector is formed and nodes nearest are taken to be the long axis $W_i^{l^1}$ and $W_i^{l^2}$ (highlighted nodes). The growth direction angle θ of cell i is the positive acute angle between the horizontal and the long axis. Aspect ratio is also calculated from the lengths of the long and short axes. Orientation is measured in the same way as for experimental images described above. Image was rendered via simulation output, and the heat map shows tensile stress calculated by node as in equation (6) in the main text.

experiments described above. Cells with aspect ratios smaller than 1.3 are considered isotropic in shape, otherwise cells are considered as anisotropically expanded along the long axis. The growth direction angle is defined as the acute angle between the x-axis and the long axis direction.

C.3. Layered organization of the epidermal L1 and L2 cell layers. To quantify the amount of deviation from the layered organization as shown in Fig 7 H, we separately calculated the root mean squared error (RMSE) of the regression lines fit to the sets of cell centers from both the L1 and L2 cell layers. The values reported in Fig 7 H are an average of the RMSE for the L1 and L2 layers across all 20 simulations for each distinct mechanism.

C.4. Dome height, width, and width-height ratios for simulations. To capture the exaggeration of the dome structure in a given meristem, we measure the *dome height* as follows. If $\{(x_j, y_j)\}$ are the centroids of all layer 1 cells, then

$$\text{dome height} := \max_j y_j - \min_j y_j.$$

To measure the SAM *width*, we measured the maximum distance between layer 1 centroids, given by the expression

$$\text{width} := \max_j x_j - \min_j x_j.$$

The width-height ratio was determined for a given SAM by calculating $\frac{\text{width}}{\text{dome height}}$.

C.5. Global curvature of the surface of the SAM. Global curvature of the surface of the SAM was used to compare the shape of the SAM after 40 hours of growth between experiments and simulations. For both experimental and simulation images, the x and y coordinates of each cell center in the L1 layer were recorded. Then a circle was fit to the resulting set of data points using the Circle Fit (Pratt method) in MATLAB. Finally, *global curvature* was calculated by taking the inverse of radius of the best fit circle for each meristem.

C.6. Spatial Distribution of WUS in the SAM. To quantify the spatial distribution of WUS in the SAM, we measured both the *radial distance* of WUS in the tissue and analyzed the number of *high WUS containing cells* in the epidermal L1 cell layer. In order to visualize the number of high WUS containing cells in the L1 layer of experimental images, we first quantified the average nuclear WUS fluorescence intensity within each cell in the L1. We then divided the average fluorescence intensity of each cell by the value of the brightest cell in its respective SAM to determine each cell's relative WUS concentration. Next, we binned the relative WUS concentration of all L1 cells by their distance (in average cell diameter) from the brightest cell (i.e. highest WUS containing cell) using a fixed bin size of the average cell diameter for the given meristem and going out to 6 average cell diameters on either side of the brightest cell. In addition, 95% confidence intervals are shown for each bin. The same process was used for simulations as demonstrated in Fig 7 L.

Similar analysis of the WUS concentration across the L2 and corpus of experimental images was used to determine the maximum diameter of the set of high WUS containing cells along the radial axis of the meristem across all layers, denoted D_{CZ}. In addition, we manually measured the diameter of the PZ, denoted D_{PZ}, for each image. We define the radial distance of WUS in the tissue as the ratio $:= D_{CZ}/D_{PZ}$ as can be seen in Fig 7 K and Fig A in S1 Appendix. In simulations, we measured the radial distance of WUS in the following way. Let $\{c_j\}$ be the set of all cell centroids for cells with WUS concentration greater than the experimentally calibrated threshold WUS_{min}. We define $D_{CZ} := \max_{i,j}\{|c_j - c_i|\}$ and D_{PZ} is equal to SAM

width, as described above. As suggested by previous studies (3), the 8 brightest cells expressing the *CLV3* reporter across the L1 encompass the stem cell population in this layer. Thus, we determined the threshold WUS_{min} for each simulation by calculating the minimum WUS value of all L1 cells within 8 average cell diameters of the highest WUS containing cell in the L1 layer.

D. Sensitivity and Perturbation Analyses on the Equilibrium State of the SAM. The equilibrium state of the model SAM, as described in section B above, is the result of stage one of the simulation rather than being controlled by any model inputs. Since the equilibrium state serves as the initial condition of the main simulation, we investigated the impact of perturbations on the initial positioning of cells as well as variations in parameters controlling the cell-wall extensibility and stiffness of boundary cells during the establishment of the equilibrium state. In these analyses, we fixed an initial selection of a growth polarization direction and number of cytoplasm nodes for each cell to remove the stochastic confounding of the analyses. We expect that for a fixed distribution of cytoplasm nodes and growth directions for each cell, any impacts clearly attributable to those fixed distributions may be averaged out since they are the only non-deterministic features of the stage one of the simulation.

We perturbed the initial positioning of each cells' center from c_i to $c_i + A \cdot \hat{v}(\Theta)$, where $\hat{v}$ is a unit vector oriented towards the uniformly randomly chosen direction Θ. We perturbed the *centroid noise amplitude* A from 0 to $0.2\mu m$, where 0 returns the unperturbed system and 0.2 is chosen to prevent cell-cell overlapping. Separately, we performed sensitivity analyses on the impacts of two mechanical parameters, $k^{\mathrm{lin}}_{\mathrm{Boundary}}$ and η_{Stem} controlling boundary behavior to assess the impact of boundary dynamics on the equilibrium structure. We examined 100 values of $k^{\mathrm{lin}}_{\mathrm{Boundary}}$ logarithmically sampled between one half and twice its default value, and sampled 100 values of η_{Stem} logarithmically around one tenth and ten times its default value. Parameter values for sensitivity and perturbation analyses were varied independently; when one was being varied, the others were fixed at their default values.

Fig C shows the results of the perturbation analysis on A for two tissue-scale and three cell-scale metrics. Though there was an increase of variance of curvature and apical surface length, the proportional increase of the curvature circle's fitness RMSE and the small absolute differences of the metric values suggest that the overall impact of the perturbation is negligible, and can be attributed to slight disruptions to the nearly-colinear centroids of the layer 1 cell layer. The cell-scale metrics show no clear impact of A on cell orientation, aspect ratio, or area distributions. Thus, we conclude that there are no critical singular perturbations of our equilibrium distribution of cells in the tissue arising from any symmetry inherent in the placement of the initial cells within the range of perturbation explored.

Fig D shows the results of the sensitivity analyses on boundary parameters $k^{\mathrm{lin}}_{\mathrm{Boundary}}$ and η_{Stem}. Within the range of perturbation we explored, the impact of η_{Stem} on all tissue-scale and cell-scale measurements were negligible, except for a slight increase in stem-layer cell area when damping values were dropped substantially. Lastly, $k^{\mathrm{lin}}_{\mathrm{Boundary}}$ was demonstrated to slightly decrease the apical surface length of the SAM by $0.3\mu m$. However, this is consistent with the observations that high-$k^{\mathrm{lin}}_{\mathrm{Boundary}}$ simulations have boundary cells shrink due to tightened walls, which forces the adhered, neighboring cells into alignment. This is further supported by the impact of $k^{\mathrm{lin}}_{\mathrm{Boundary}}$ cell area, wherein the equilibrium area of a boundary is smaller with a higher value of $k^{\mathrm{lin}}_{\mathrm{Boundary}}$. For all non-boundary cells, the impact of $k^{\mathrm{lin}}_{\mathrm{Boundary}}$ is negligible.

E. CAE-M Division Plane Mechanism in 3D. In what follows, we describe how the CAE-M division plane mechanism would be implemented in a 3D, subcellular element (SCE) model of cells of the SAM (see Fig E). In our 3D model of cells of the SAM, plant cell walls are discretized using a triangular mesh consisting of a set of edges and nodes in three dimensions. The 3D version of the CAE-M mechanism of positioning the division plane consists of first calculating the local stresses at each cell wall node in the plane tangent to the cell wall surface at each node. We denote the tangent plane at node N to be T_N. The cell wall node with the maximal net stress (i.e. the node with the largest sum-square of stresses projected onto T_N) is chosen as the "base-point" or node with maximal stress denoted N_{Max}. Next, we compute the local stress vectors between cell wall nodes in a small neighborhood around N_{Max} and then project these stresses onto $T_{\mathrm{N_{Max}}}$. The vector v_{max} is chosen as the average of these projected stresses that represents the maximal stress direction on the cell surface at N_{Max}, then we find a vector v_1, which is orthogonal to v_{max} and in the plane $T_{\mathrm{N_{Max}}}$. At this point, candidate division planes are any planes which include both the vector v_1 and the point N_{Max}, and so we choose the 3D CAE-M division plane to be the one that also divides the cell volume approximately in half. Note that if our calculation results in $v_1 = 0$, this represents isotropic local stress and in this case v_1 is selected as a random direction in $T_{\mathrm{N_{Max}}}$. We included Fig F below to demonstrate that, in at least a simplified case, the division plane line segment in 2D lies at the intersection of the 3D division plane and the longitudinal SAM section we are modeling in simulations.

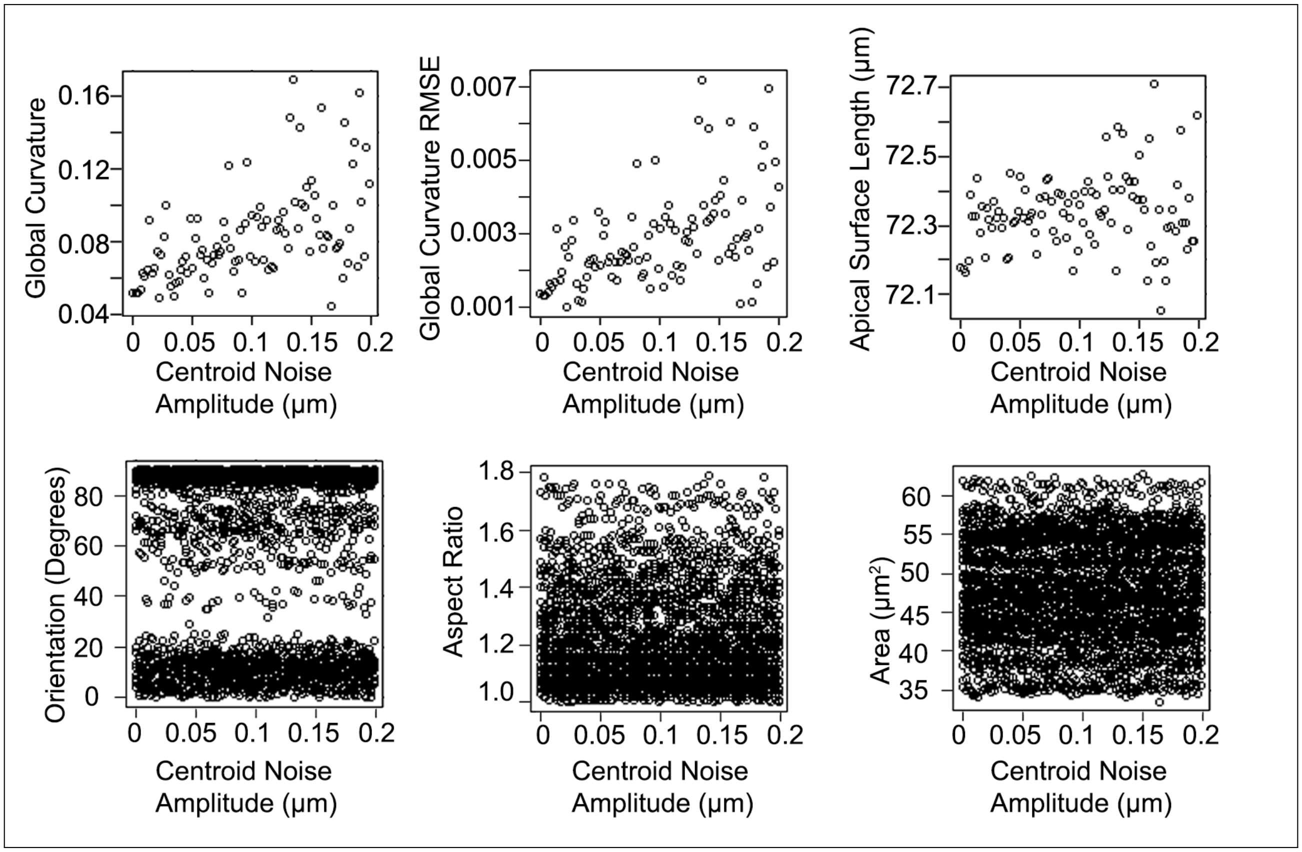

Fig. C. Perturbation Analysis of Equilibrium State. Perturbation analysis results of adding small, random displacements to initial cell locations on the mechanical equilibrium state of the tissue - i.e. after representing t =15 minutes of growth, as discussed in section D. The top left and top center panels demonstrate both the impact of cell center displacement on global curvature and how that impact may be attributed to the fitness of a circle to the surface. The top right panel shows that the apical surface length of the equilibrium SAM is only impacted via a negligible increase in variation of the total apical surface length. The bottom panels show all cell-level measurements of non-boundary cell orientation, aspect ratio, and areas from 100 simulations of increasing centroid noise amplitude. These panels exhibit independence of cell geometry and orientation from random cell displacement - suggesting that the initial tiling's precise spatial arrangement does not play a role in the equilibrium distribution of cells.

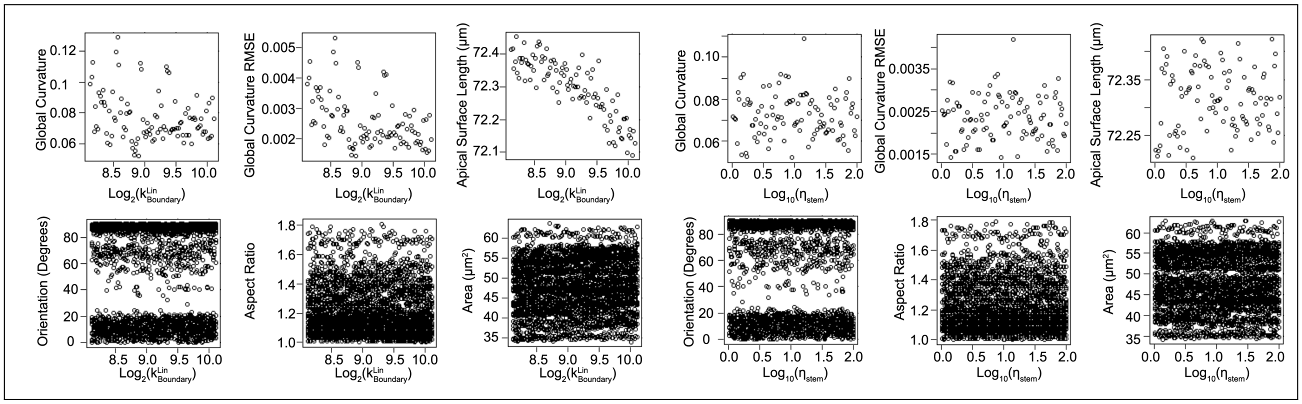

Fig. D. Sensitivity Analysis of Equilibrium State. Tissue scale (top) and cell-scale (bottom) measurements taken from simulated SAMs after 15 minutes of simulated growth were represented as in section D. Values of k^{lin} (left 6 panels) and η_{Boundary} (right 6 panels) were independently varied to 100 values around their default. (Top) all tissue-scale impacts on boundary dynamics were negligible, with the exception of the reduction of apical surface length with a fourfold increase of $k^{\text{lin}}_{\text{Boundary}}$. However, this is attributable to the shrinkage of the boundary cells with high k^{lin} on the boundary which, upon observation, appear to pull the SAM surface flatter. This difference is less than half of a micron, and so we conclude that the overall impact of boundary cell properties on tissue-scale measurements is negligible. (Bottom) Local sensitivity analysis shows no impact of passive boundary mechanical properties on equilibrium cell shape.

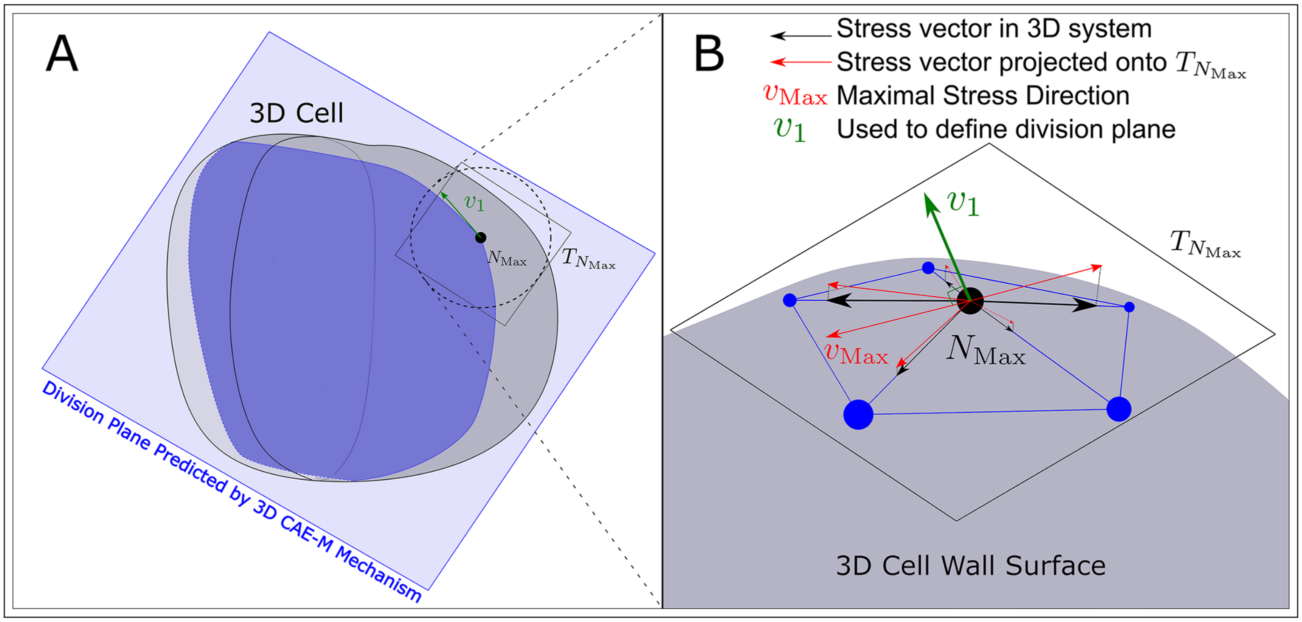

Fig. E. CAE-M division plane mechanism in 3D model. (A) Diagram of a 3D model cell is shown in gray and depicts the cross-section of the division plane predicted by the CAE-M mechanism in blue. The vector v_1 (green) is used to determine the cell division plane by finding the plane that includes both v_1 and N_{Max}, and that divides the cell volume approximately in half. (B) Diagram of the calculation of v_{Max}, the maximal stress direction in $T_{N_{\text{Max}}}$ within a neighborhood of N_{Max}, and the resulting vector v_1 in $T_{N_{\text{Max}}}$ orthogonal to v_{Max}. Vectors indicating stresses acting on N_{Max} as a result of nearby cell wall nodes are indicated in black, and their projection onto the tangent plane $T_{N_{\text{Max}}}$ is shown in red.

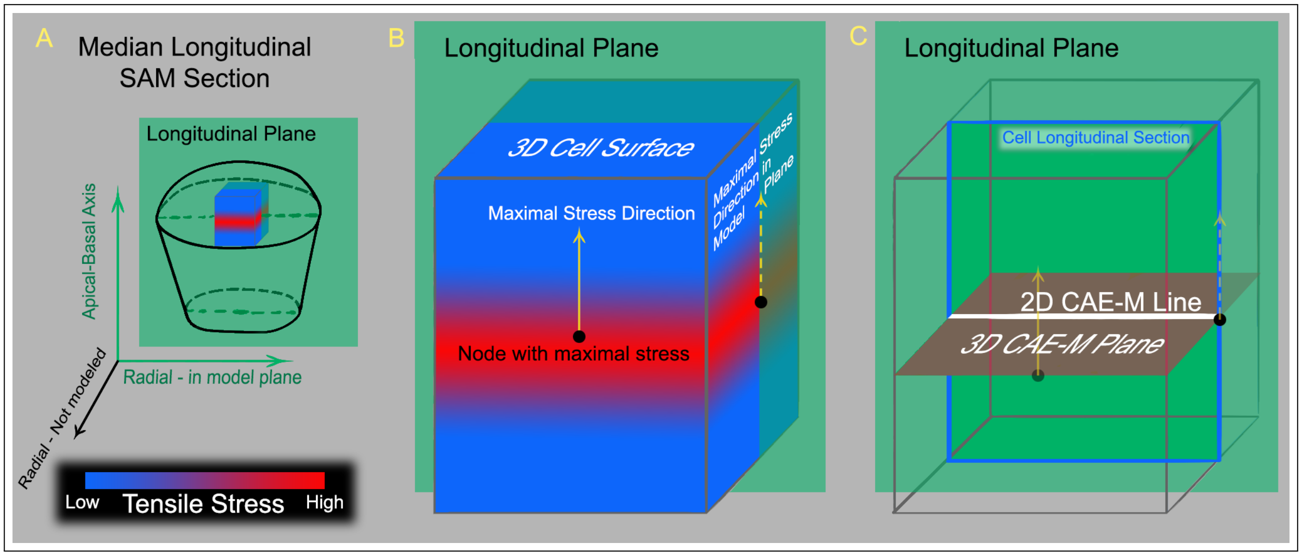

Fig. F. CAE-M division plane in 3D cell and corresponding 2D model plane. (A) 3D SAM (black lines) with cell (blue) intersected by the 2D longitudinal plane (green) our model simulates. (B) 3D cell (blue) intersected by the 2D model plane (green). The front face of the cell shows the out-of-plane node with maximal stress and maximal stress direction (solid yellow arrow) found using the 3D version of our CAE-M mechanism. The right face of the cell shows the in-plane node with maximal stress and maximal stress direction (dashed yellow arrow) found using the 2D version of our CAE-M mechanism based on in-plane tensile stresses. (C) 3D cell intersected by 2D model plane (green) and the division plane predicted by the 3D CAE-M mechanism (brown). The line segment predicted by the 2D CAE-M mechanism lies at the intersection of the 3D division plane and the 2D model plane (solid white line).

References

1. M Banwarth-Kuhn, et al., Cell-Based Model of the Generation and Maintenance of the Shape and Structure of the Multilayered Shoot Apical Meristem of Arabidopsis thaliana. *Bull. Math. Biol.* **81**, 3245–3281 (2019).
2. M Banwarth-Kuhn, Ph.D. thesis (University of California, Riverside) (2019).
3. M Perales, et al., Threshold-dependent transcriptional discrimination underlies stem cell homeostasis. *Proc. Natl. Acad. Sci. U. S. A.* **113**, E6298–E6306 (2016).
4. P Martinez, et al., Predicting division planes of Three-Dimensional cells by Soap-Film minimization. *Plant Cell* **30**, 2255–2266 (2018).
