# Supplementary material for: Combined computational modeling and experimental analysis integrating chemical and mechanical signals suggests possible mechanism of shoot meristem maintenance
Source: PLoS Comput Biol. 2022 Jun 21;18(6):e1010199. doi: 10.1371/journal.pcbi.1010199 (PMC9249181; doi:10.1371/journal.pcbi.1010199)
Supplement: S1 Fig — Consecutive periclinal division lead to the formation of strips in wildtype (black) and ectopic misexpression of cytokinin (red). 4-cell strips are caused by three sequential periclinal divisions and 10-cell strips are caused by nine sequential periclinal division. Significance was determined by student T-test for ectopic misexpression of CK signaling compared to wildtype. Asterisks indicate significance at the following levels ****p < 0.0001. (PDF) [file pcbi.1010199.s004.pdf]

1 **S1. Fig**

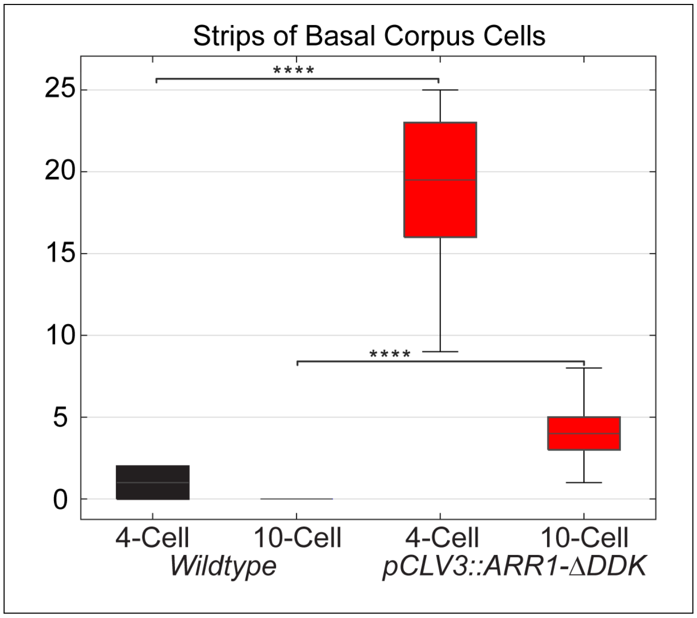

Fig. S1. Cytokinin signaling increases periclinal cell divisions in Basal Corpus. Consecutive periclinal division lead to the formation of strips in wildtype (black) and ectopic misexpression of cytokinin (red). 4-cell strips are caused by three sequential periclinal divisions and 10-cell strips are caused by nine sequential periclinal division. Significance was determined by student T-test for ectopic misexpression of CK signaling compared to wildtype. Asterisks indicate significance at the following levels ****$p < 0.0001$.
