# Supplementary material for: Combined computational modeling and experimental analysis integrating chemical and mechanical signals suggests possible mechanism of shoot meristem maintenance
Source: PLoS Comput Biol. 2022 Jun 21;18(6):e1010199. doi: 10.1371/journal.pcbi.1010199 (PMC9249181; doi:10.1371/journal.pcbi.1010199)
Supplement: S2 Fig — The distributions of cell orientations for mother (solid lines) and daughter cells (dashed lines) in the (A) apical corpus and (B) basal corpus. The distributions of (C) cell heights and (D) cell widths in the apical corpus. The distribution of (E) cell heights and (F) cell widths in the basal corpus. In all Fig, experimental data (black), CAE-E (blue), CAE-M (gold), and CED(green). Significance was determined by Levene’s test. (PDF) [file pcbi.1010199.s005.pdf]

# S2. Fig

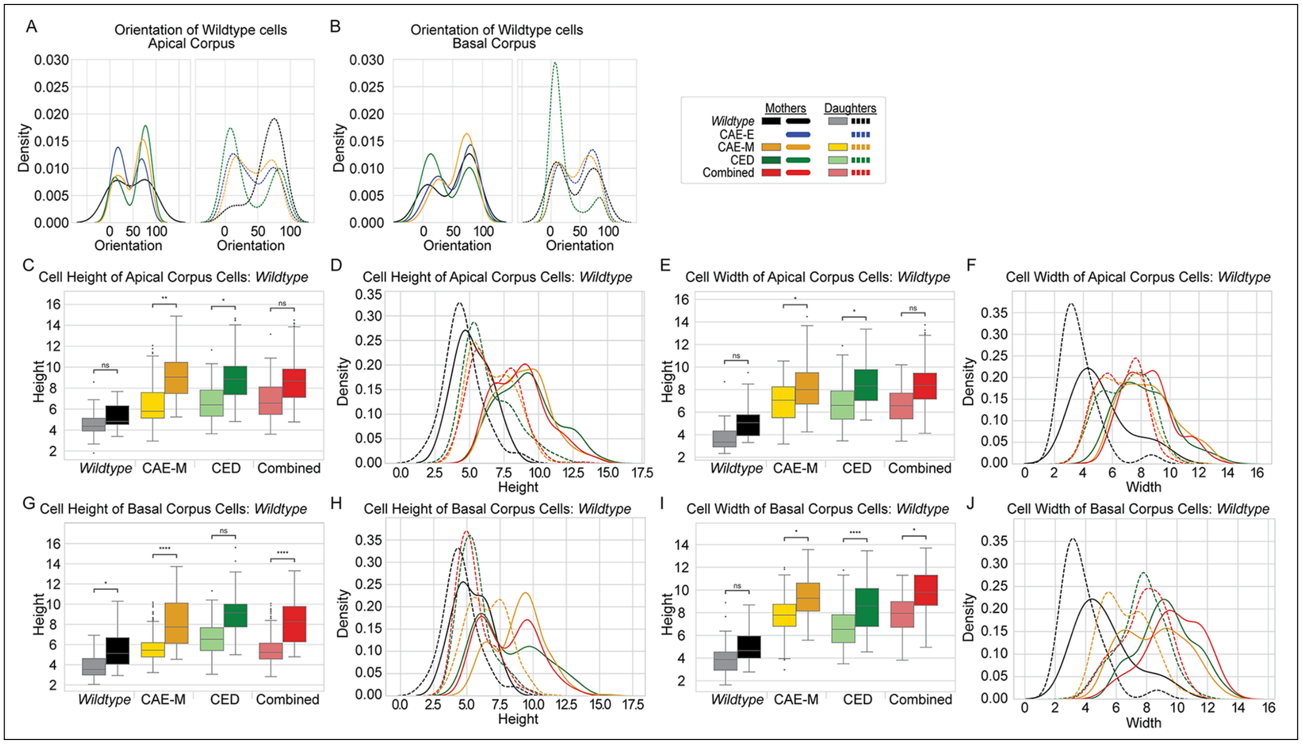


**Fig. S2. Comparison of cell orientations, heights, and widths between experimental wildtype SAMs and wildtype simulations for all four hypothesized mechanisms.** The distributions of cell orientations for mother (solid lines) and daughter cells (dashed lines) in the (A) apical corpus and (B) basal corpus. The distributions of (C) cell heights and (D) cell widths in the apical corpus. The distribution of (E) cell heights and (F) cell widths in the basal corpus. In all Fig, experimental data (black), CAE-E (blue), CAE-M (gold), and CED(green). Significance was determined by Levene's test.
