# Supplementary material for: Combined computational modeling and experimental analysis integrating chemical and mechanical signals suggests possible mechanism of shoot meristem maintenance
Source: PLoS Comput Biol. 2022 Jun 21;18(6):e1010199. doi: 10.1371/journal.pcbi.1010199 (PMC9249181; doi:10.1371/journal.pcbi.1010199)
Supplement: S4 Fig — The distribution of (A) cell aspect ratios and (B-D) orientations in the apical corpus and basal corpus from ectopic misexpression of WUS in experiments [pCLV3::LhG4; 6xOP;eGFP-WUS-GR] and simulations comparing three hypothesized mechanisms. (A,B) The distribution for all cells in experimental (black), CAE-E (blue), CAE-M (gold), and CED (green). The distribution of cell orientations for (C) mother cells (solid lines) and (D) daughter cells (dashed lines) were segregated based on cell size and independently graphed. The distributions of (E-F) cell heights and (G-H) cell widths in the apical corpus of cells in ectopic misexpression of WUS condition. The distributions of (I-J) cell heights and (K-L) cell widths in the basal corpus of cells in ectopic misexpression of WUS condition. (M) Amount of deviation from a single-cell layer in the epidermal L1 and L2 cell layers for experimental SAMs (black), CAE-E simulations (blue), CAE-M simulations (gold), and CED simulations (green) in the ectopic misexpression of WUS condition. (N) The ratio of SAM width to dome height for experimental SAMs (black), CAE-E simulations (blue), CAE-M simulations (gold), and CED simulations (green) in the ectopic misexpression of WUS condition. (O) Global curvature of the SAM surface for experimental SAMs (black), CAE-E simulations (blue), CAE-M simulations (gold), and CED simulations (green) in the ectopic misexpression of WUS condition. See S3 Appendix for detailed description of all metrics used in this Figure. (PDF) [file pcbi.1010199.s007.pdf]

S4. Fig

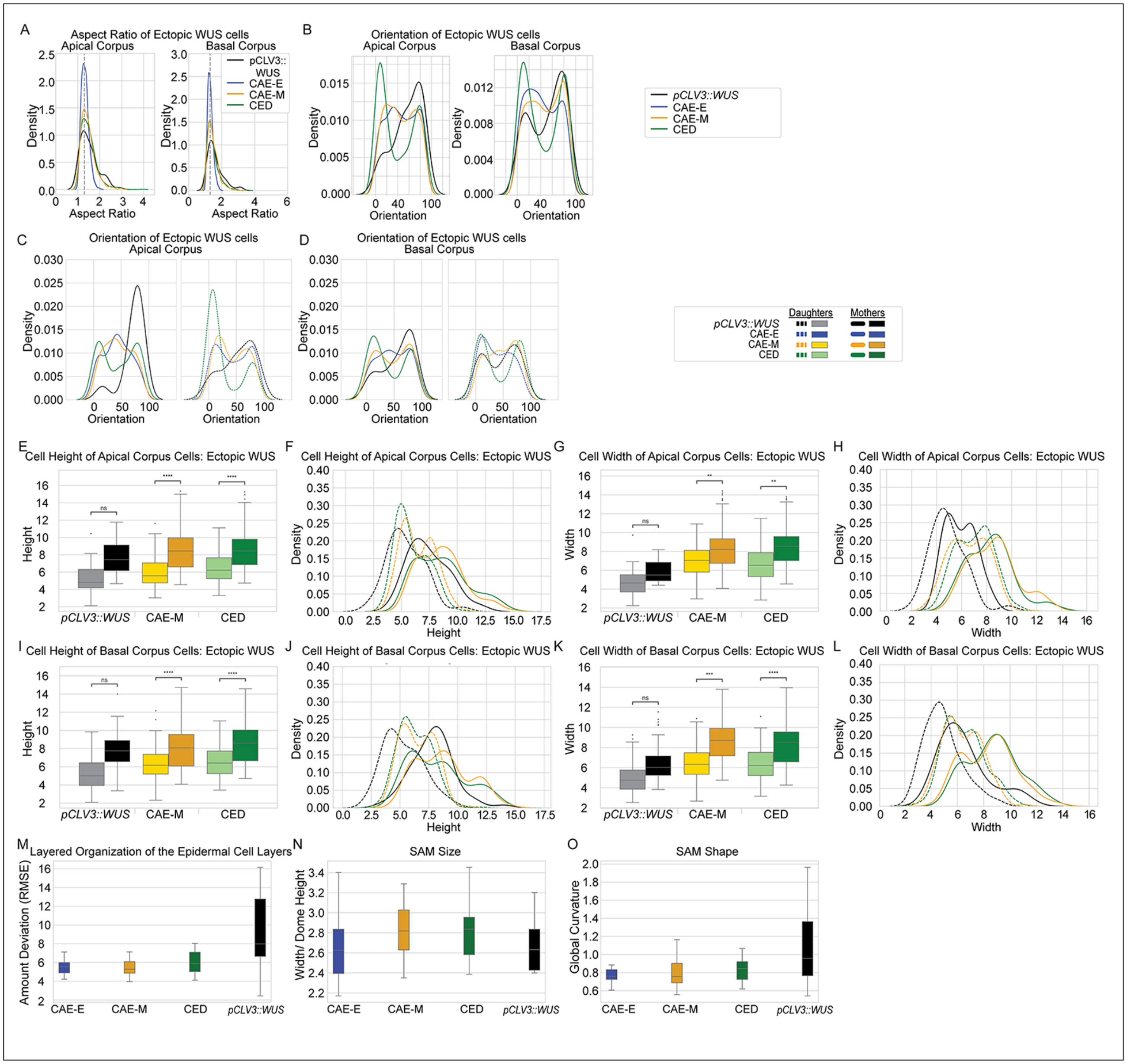

Fig. S4. Ectopic misexpression of WUS influences the direction of anisotropic cell expansion. The distribution of (A) cell aspect ratios and (B-D) orientations in the apical corpus and basal corpus from ectopic misexpression of WUS in experiments [pCLV3::LhG4; 6xOP;eGFP-WUS-GR] and simulations comparing three hypothesized mechanisms. (A,B) The distribution for all cells in experimental (black), CAE-E (blue), CAE-M (gold), and CED (green). The distribution of cell orientations for (C) mother cells (solid lines) and (D) daughter cells (dashed lines) were segregated based on cell size and independently graphed. The distributions of (E-F) cell heights and (G-H) cell widths in the apical corpus of cells in ectopic misexpression of WUS condition. The distributions of (I-J) cell heights and (K-L) cell widths in the basal corpus of cells in ectopic misexpression of WUS condition. (M) Amount of deviation from a single-cell layer in the epidermal L1 and L2 cell layers for experimental SAMs (black), CAE-E simulations (blue), CAE-M simulations (gold), and CED simulations (green) in the ectopic misexpression of WUS condition. (N) The ratio of SAM width to dome height for experimental SAMs (black), CAE-E simulations (blue), CAE-M simulations (gold), and CED simulations (green) in the ectopic misexpression of WUS condition. (O) Global curvature of the SAM surface for experimental SAMs (black), CAE-E simulations (blue), CAE-M simulations (gold), and CED simulations (green) in the ectopic misexpression of WUS condition. See S2 Text for detailed description of all metrics used in this Fig
