# Supplementary material for: Combined computational modeling and experimental analysis integrating chemical and mechanical signals suggests possible mechanism of shoot meristem maintenance
Source: PLoS Comput Biol. 2022 Jun 21;18(6):e1010199. doi: 10.1371/journal.pcbi.1010199 (PMC9249181; doi:10.1371/journal.pcbi.1010199)
Supplement: S5 Fig — The distributions of cell orientations (Left) and aspect ratios (Right) at various time points computed directly from simulations, organized by cell division plane mechanisms and perturbation condition. The distributions of cell orientations and aspect ratios were obtained for the combined mechanism only in the wildtype simulations. (PDF) [file pcbi.1010199.s008.pdf]

S5. Fig

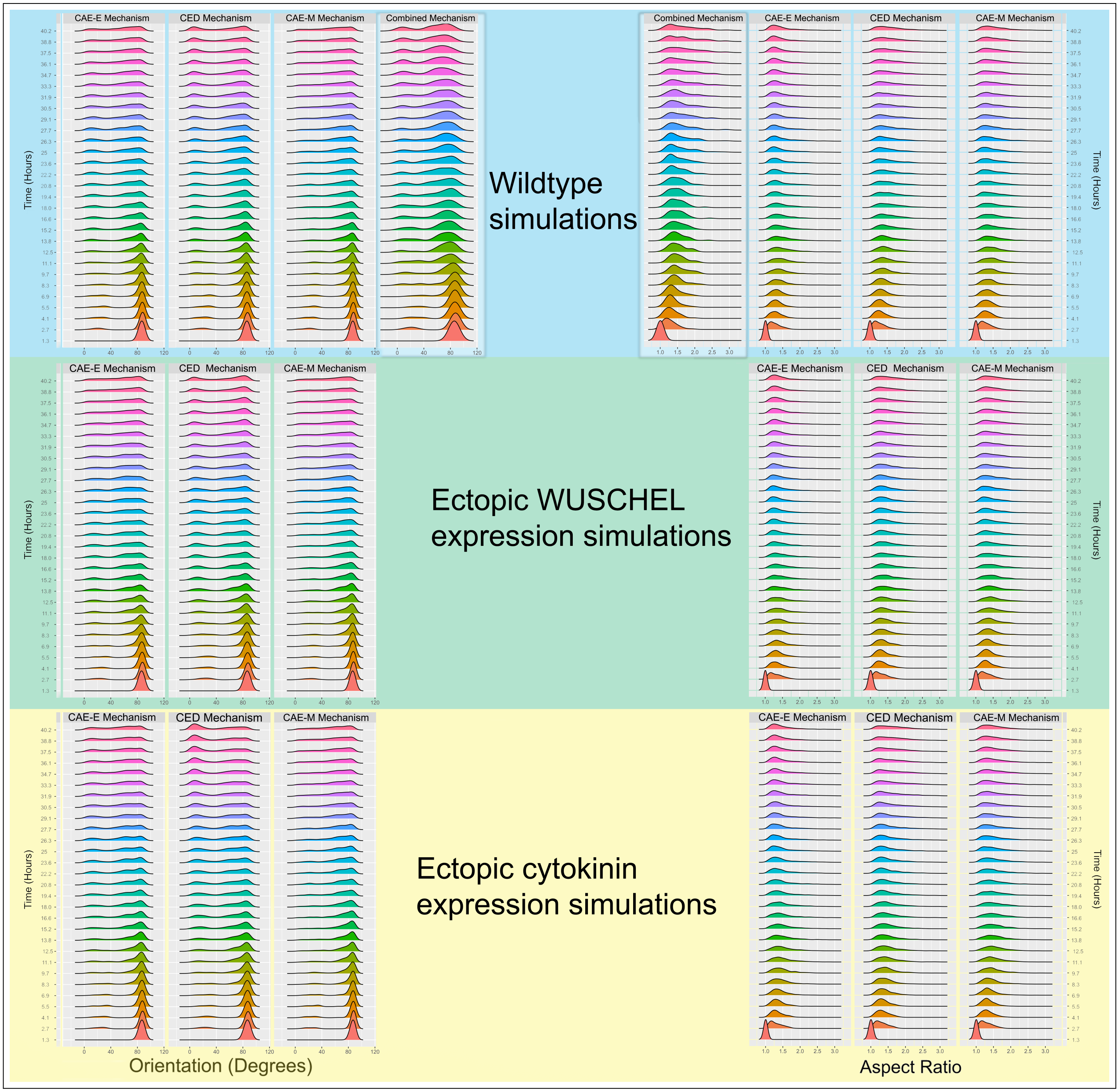

Fig. S5. Time evolution of simulated cell orientations and aspect ratios by condition and division plane mechanism. The distributions of cell orientations (Left) and aspect ratios (Right) at various time points computed directly from simulations, organized by cell division plane mechanisms and perturbation condition. The distributions of cell orientations and aspect ratios were obtained for the combined mechanism only in the wildtype simulations.
