# Supplementary material for: Combined computational modeling and experimental analysis integrating chemical and mechanical signals suggests possible mechanism of shoot meristem maintenance
Source: PLoS Comput Biol. 2022 Jun 21;18(6):e1010199. doi: 10.1371/journal.pcbi.1010199 (PMC9249181; doi:10.1371/journal.pcbi.1010199)
Supplement: S6 Fig — (A) Aspect ratio and (B) orientation of L1 and L2 cells in from wildtype, ectopic misexpression of WUS [pCLV3::LhG4; 6xOP::eGFP-WUS-GR], and ectopic misexpression of CK [pCLV3::LhG4; 6xOP::ARR1-ΔDDK-GR] experimental SAMs. (C-F) Cell layer specific aspect ratio and orientation of cells from wildtype, wus1 mutants [wus1–1], and cytokinin triple receptor mutants[cre1;ahk2;ahk3]. Cell height (G) and width (H) of L1 and L2 cell layers for each experimental condition. Significance was determined by t-test for each experimental condition compared to wildtype. Asterisks indicate significance at the following levels:****p < 0.0001. (PDF) [file pcbi.1010199.s009.pdf]

S6. Fig

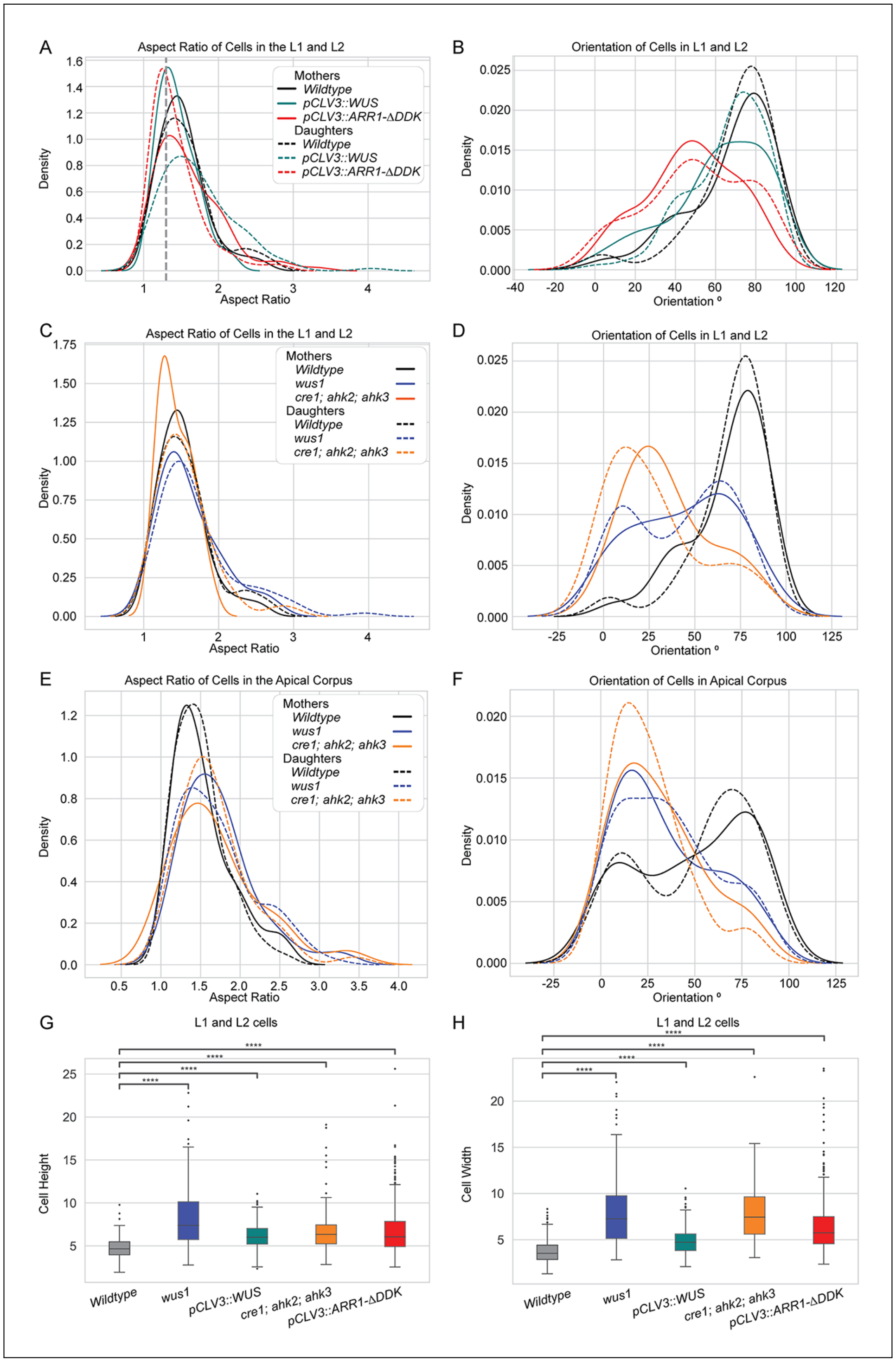

Fig. S6. WUS and CK misexpression and loss of function mutants influence the direction of anisotropic expansion of cells. (A) Aspect ratio and (B) orientation of L1 and L2 cells in from wildtype, ectopic misexpression of WUS [pCLV3::LhG4; 6xOP::eGFP-WUS-GR], and ectopic misexpression of CK [pCLV3::LhG4; 6xOP::ARR1-ΔDDK-GR] experimental SAMs. (C-F) Cell layer specific aspect ratio and orientation of cells from wildtype, *wus1* mutants [*wus1-1*], and cytokinin triple receptor mutants[*cre1;ahk2;ahk3*]. Cell height (G) and width (H) of L1 and L2 cell layers for each experimental condition. Significance was determined by t-test for each experimental condition compared to wildtype. Asterisks indicate significance at the following levels:****p $<$ 0.0001.
